# Developing and evaluating a multidimensional progress-based assessment standard for standing long jump performance in university physical education: a sequential mixed-methods study

**DOI:** 10.3389/fpsyg.2026.1861330

**Published:** 2026-08-12

**Authors:** Wenyu Zhang, Fengmin Zhang, Haipeng Yang, Xiaofeng Lin

**Affiliations:** 1 School of Physical Education, Minzu University of China, Beijing, China; 2 Faculty of Education, Universiti Kebangsaan Malaysia, Bangi, Selangor, Malaysia

**Keywords:** evaluation criteria, learning motivation, multidimensional evaluation, physical education assessment, sequential mixed-methods

## Abstract

Physical education assessments that count toward university admission or course grades typically measure only a student’s fitness at a single time point, overlooking incremental progress and thus providing little incentive for those with lower baseline performance. This study evaluated the feasibility of a multidimensional scoring method that captures changes in standing long jump performance, an indicator of lower-limb explosive power and better reflects student learning. This study had two phases. Phase 1 combined a two-round Delphi process, AHP weighting, and standing long jump data from 211 classes (*N* = 4,109) to develop the multidimensional evaluation model. Phase 2 is a quasi-experimental study where 4 year 1–2 university classes was randomly selected and assigned to the intervention (IG) and control group (CG). Learning motivation was measured using the college student version of the Work Preference Inventory. Difference-in-difference model was used to analyze the association between motivation and standing long jump distance (post–pre) between IG and CG. Both IG and CG showed significant improvements in both standing long jump and motivation, with IG having significantly better outcomes. The time interaction between the groups confirmed the statistical significance. The OLS regression results showed both internal motivation (*p* < 0.001) and external motivation (*p* < 0.001) had significant positive relationship. Both boys’ (*p* < 0.001) and girls’ (*p* = 0.029) showed significant improvement. These findings suggest that a multidimensional, progress-sensitive scoring system can enhance students’ motivation and foster positive behavioral change in physical education.

## 1 Introduction

In recent years, physical education in higher education has increasingly become a focus of scholarly and public attention, particularly with respect to the effectiveness and fairness of student performance assessment. *The Overall Plan for Deepening Education Evaluation Reform in the New Era (China, 2020)* emphasizes the need to move beyond unscientific evaluation practices (Taber, 2018; Saaty, 1980). In this context, single-dimensional assessment models based solely on absolute scores are insufficient. A more comprehensive approach that considers both performance and effort is essential to enhance student motivation and support the long-term development of college physical education (Arik and Erturan, 2023; Baena-Morales et al., 2024; Su et al., 2022). In contrast to other academic subjects, physical education is characterized by considerable variation in students’ physical fitness, which makes it essential to address this diversity comprehensively when seeking to enhance their motivation for participation (Ezeddine et al., 2023). Tendinha et al. (2021) demonstrated that students’ motivation for physical activity is shaped by effort, enjoyment, and active participation. Tariq and Sergio (2025) emphasized that diversified evaluation fosters students’ learning interest and motivation while guiding evaluators to focus on participation, skill development, and health literacy. Although recent reforms in university physical education in China have improved students’ performance and motivation to some extent, the evaluation framework remains single-dimensional and requires further enrichment and refinement (Li and Zhang, 2024). According to Ferriz Valero et al. (2023), learning outcomes in physical education —given its dual emphasis on technical skills and physical fitness—are shaped by both acquired effort and inherent physical capacity. Due to substantial differences in students’ baseline levels, the difficulty and extent of progress under identical teaching objectives are uneven. Students with higher baseline levels face greater challenges in reaching advanced performance, whereas those with lower levels often struggle to keep pace with the course (Campbell, 2022). In practice, teachers often set teaching difficulty and exercise intensity at a moderate level to ensure that most students meet course requirements. However, this approach limits exercise opportunities for high-performing students, while those with lower abilities struggle to make consistent progress (Fahrizqi et al., 2021). In addition, specialized course teaching often prioritizes technical skills over physical fitness, a neglect that undermines the foundational role of physical training and ultimately weakens skill development outcomes (Ribeiro et al., 2021). Physical fitness accounts for about 30–40% of the total physical education score in colleges and universities (Chen et al., 2022), Therefore, good physical reserve is a prerequisite for students to master special skills (Annenkova et al., 2021). However, physical fitness evaluation continues to rely on a single absolute standard, disregarding individual differences in effort and learning attitude. Such a narrow approach not only limits the accuracy of assessment but also fails to reflect the broader development of students’ comprehensive qualities (Li and Zhang, 2024).

Based on Self-Determination Theory (SDT) (Deci and Ryan, 1985), physical education assessment should not focus solely on end-of-term “outcome scores.” Instead, assessment design should be used to provide students with a more supportive learning environment. SDT posits that improvements in learning motivation depend on students’ experiences of autonomy, competence, and relatedness support during learning. When an assessment system emphasizes progress over time, articulates clear criteria, and provides ongoing feedback, students are more likely to perceive a controllable link between their effort and their performance. This perception can strengthen students’ competence and increase their willingness to engage persistently (Deci and Ryan, 1985). Accordingly, recent research on physical education assessment has gradually moved beyond a sole reliance on “absolute achievement” and has increasingly focused on students’ growth trajectories and process-oriented development. This shift has generated extensive work on progress-based assessment, formative assessment, and multidimensional scoring rubrics or composite indices (Alasqah, 2022; Black and Wiliam, 1998). Progress-based assessment incorporates students’ improvement toward predefined goals as a core indicator, thereby alleviating the unfairness of traditional one-off tests for students with low baseline performance. Classroom intervention studies further indicate that when assessment criteria shift from end-of-term absolute scores to staged incremental performance, students with lower starting points are more likely to demonstrate higher classroom participation, stronger self-efficacy, and greater willingness to practice consistently. At the same time, teachers can more readily identify students who require additional support and provide differentiated guidance. Meanwhile, quantitative research grounded in Self-Determination Theory (SDT) provides a coherent theory-to-evidence chain explaining why a multidimensional, progress-based assessment can enhance students’ learning motivation. A multilevel study found that clearer knowledge of the criteria for an upcoming physical education assessment was associated with higher situational autonomous motivation and lower maladaptive motivation, and these associations were largely accounted for by satisfaction of the needs for autonomy, competence, and relatedness (Haerens et al., 2019). Likewise, a structural equation modeling study showed that the implementation level of assessment for learning was positively related to students’ basic psychological need satisfaction and higher-quality autonomous motivation, which in turn predicted greater effort investment and better course grades (Ulstad et al., 2025). Although the two studies emphasize different aspects, they converge on the same mechanism: when assessment information is more transparent and usable, students are more likely to experience competence and self-regulatory control, thereby sustaining effort investment and more adaptive motivational states. Together, these SDT-based quantitative findings yield a testable theoretical expectation for assessment reform: greater transparency, progress-referenced criteria, and formative feedback are more likely to foster improvements in motivation and engagement. The remaining challenge is that, although prior research has documented a broader shift from an exclusive focus on absolute scores toward approaches that jointly value progress and formative evidence, relatively limited work has structurally integrated progress-based assessment with absolute performance within university physical fitness evaluation and validated such models empirically.

To address the limitations of existing single-dimensional assessment criteria, this study used the standing long jump unit in university physical education as the instructional context to develop a multidimensional assessment standard. The proposed standard was therefore intended as a task-specific proof of concept for standing long jump assessment rather than as a comprehensive standard for university physical education. The study employed a sequential mixed-methods development-and-evaluation design to complete a two-phase “development–evaluation” process and examined the effects of the proposed standard on students’ learning motivation and physical performance in authentic classroom settings. This study hypothesized that the multidimensional assessment standard would enhance students’ learning motivation and test performance, thereby providing empirical evidence for progress-based standing long jump assessment in higher education. From a motivational perspective, SDT suggests that learners’ autonomous motivation is more likely to be activated when students’ needs for autonomy and competence are satisfied (Ryan and Deci, 2000). When assessment criteria are referenced to individual progress and assessment rules are transparent, students can continuously receive informational feedback linked to their effort. Students can also more clearly perceive a controllable relationship between “effort, improvement, and performance gains,” which strengthens competence and facilitates motivation internalization. This mechanism is particularly important in physically demanding tasks with substantial baseline differences among students, because progress-based assessment can reduce low-baseline students’ frustration and withdrawal risk under a traditional “absolute performance” framework. Based on these theoretical arguments, this study conceptualized multidimensional progress-based assessment as a motivation-supportive instructional intervention. Accordingly, the study proposed that implementing this assessment system would increase students’ learning motivation and further promote improvements in physical performance. The intervention tool in this study was a “multidimensional progress-based assessment standard.” Its core purpose was not to include “motivation” as a scoring dimension directly in grade calculation. Instead, the tool aimed to reshape students’ learning experiences and effort-regulation strategies through explicit progress-oriented scoring rules and a transparent feedback mechanism.

Accordingly, this study pursued three research objectives: (1) to develop a multidimensional, progress-referenced assessment standard for the standing long jump unit in university physical education; (2) to examine whether implementation of the proposed standard increases students’ intrinsic and extrinsic motivation; and (3) to evaluate whether the standard improves standing long jump performance.

## 2 Study design

This study adopted an exploratory sequential mixed-methods design, consisting of three linked stages—development, implementation, and evaluation—to ensure clear methodological coherence between constructing the multidimensional assessment system and testing its effects. The project was conducted from September 2022 to January 2025. Study 1 (September 2022 to July 2023) focused on developing the multidimensional progress-based assessment standard, whereas Study 2 (September 2023 to July 2024) examined the educational effects of the standard in authentic classroom settings. Prior to the commencement of the study, written informed consent was obtained from all participants, and ethical approval was granted by the Biological and Medical Ethics Committee of Minzu University of China (ECMUC2022010CO).

The standing long jump was selected as the indicator of physical performance based on two considerations: feasibility for classroom implementation and theoretical relevance for measurement. First, the standing long jump is a field-based test that reflects lower-limb explosive power. The test demonstrates adequate validity and reliability and is suitable for convenient and stable monitoring of students’ changes in physical capability in large-class contexts (Marin-Jimenez et al., 2024). Second, the standing long jump is a commonly used indicator in China’s student physical fitness monitoring system. The indicator aligns well with the local assessment context and is practically operable. Standardised testing can be completed under limited class time and facility constraints, with relatively low organizational burden. Accordingly, this study treated the standing long jump as a task-specific indicator of lower-limb explosive power to evaluate the impact of the multidimensional progress-based assessment intervention on students’ physical performance.

In Study 1, the Delphi method was first used to screen and refine the assessment dimensions and indicators, and the assessment framework was established based on expert consensus. The analytic hierarchy process (AHP) was then used to assign weights to each dimension. The study further developed corresponding scoring rules and a composite index by considering the characteristics of assessment in physical education classes. Therefore, the final output of Study 1 included not only the “dimensions and weights” but also a classroom-ready multidimensional assessment toolkit. The toolkit comprised scoring formulas, weighting coefficients, grading rules, and implementation guidelines, and it served as the core instructional intervention in Study 2.

In Study 2, the multidimensional assessment toolkit developed in Study 1 was embedded into assessment practice within a university physical education course. A class-level cluster-randomized quasi-experimental design was used to allocate classes to conditions and implement the intervention. The intervention group used the multidimensional assessment standard for formative feedback and grade calculation, whereas the control group adopted the conventional assessment approach. To estimate the intervention effect, this study used Difference-in-Differences (DID) to compare changes in outcomes before and after the intervention, thereby identifying the treatment effect of introducing the multidimensional assessment standard on students’ learning motivation and physical performance (Abadie, 2018). DID relies on the identifying assumption that, in the absence of the intervention, the outcome trends of the two groups would have been similar. Given the two-wave design, this study primarily strengthened inferential credibility through baseline balance checks and robustness analyses (Baker et al., 2025). Because only one pre-intervention measurement was available, a formal pre-trend test could not be conducted. Therefore, baseline comparability tests and group-mean trajectory plots were used as supplementary evidence for the identifying assumption, and class-level cluster-robust standard errors were applied in the regression models.

### 2.1 Study 1

#### 2.1.1 Participants

In study 1, the participants were divided into two parts, including experts and students: (1) Fifteen experts in physical education measurement and evaluation in China were purposively selected to participate in determining the dimensions and weights of the evaluation criteria. The expert inclusion criteria were as follows: (a) at least 10 years of professional or research experience in relevant fields, such as physical education assessment and educational measurement and evaluation; (b) an academic qualification of associate professor or above; and (c) prior experience in university physical education teaching, assessment system design, or related scholarly publications. In total, 15 experts who met these criteria participated in the study. The panel’s professional expertise covered university physical education teaching, assessment-system design, and related research. (2) Students were selected from undergraduate students who usually attend public physical education courses at Minzu University of China. This school has a relatively comprehensive public physical education curriculum system and a standardized physical education testing platform, which ensures the consistency of experimental interventions and measurements. The student body includes 55 ethnic minorities, such as Han, Mongolian, Tibetan, and Uyghur, and ethnic minority students account for approximately 50% of the total student population. Students are widely distributed across 32 provinces in China. The varied composition of the sample contributes to improving the robustness of the study’s findings. The participants in this study were first- and second-year undergraduate students who usually attend public physical education courses at Minzu University of China. Boys (*n* = 1888) and girls (*n* = 3,091) participated in the pre-test, while boys (*n* = 1916) and girls (*n* = 3,068) participated in the post-test. Additionally, boys (*n* = 1,479) and girls (*n* = 2,630) participated in both tests. The total number of people whose data were finally used in Study 1 was 4,109.

#### 2.1.2 Method

Lund and Kirk (2019), it is proposed to introduce progress evaluation into physical education evaluation, enriching the evaluation dimensions, which is conducive to improving students’ motivation and collecting more accurate evaluation results. By collecting and creating dimensions, it aims to improve physical education learning motivation in all aspects better and promote physical exercise. In Study 1, the weighted summation method is used to construct the evaluation criteria. The weighted summation method is a statistical analysis technique that assigns weights according to the importance of each indicator and then sums the indicator scores based on these weights to conduct the evaluation. To combine dimensions and weights, a multi-dimensional evaluation criterion is ultimately formed. The weighted summation method is selected as the basis for this study and the construction of evaluation criteria:


$$\sum_{i=1}^{n}w_{i}p_{\mathrm{ij}}=w_{1}p_{1j}+w_{2}p_{2j}+\cdots \cdots +w_{n}p_{\mathrm{nj}}$$

Where $\sum_{i=1}^{n}w_{i}p_{\mathrm{ij}}$ represents the total score of the j evaluation component; $w_{1},w_{2},\cdots ,w_{n}$ are the weight coefficients of each evaluation component; $p_{1j},p_{2j},\cdots ,p_{\mathrm{nj}}$ are the scores of n evaluation indicators on the j component.

First, determine whether the dimension is feasible. According to Zhu et al. (2024), the importance of several dimensions is scored. The preset consistency standard usually determines the dimensions. Study 1 uses the Delphi method to determine the dimensions of this study. Using a 5-point Likert scale, the evaluation experts select each dimension and can also provide feedback on the dimension selection and other issues. Two rounds of Delphi consultation were conducted. In Round 1, experts independently rated the proposed assessment dimensions and indicators and provided revision suggestions. Based on this feedback, the research team refined the wording and structure of the indicators and merged or removed items that were redundant or unclear. In Round 2, experts re-rated the revised framework to examine the stability and consistency of expert judgments. Through this procedure, the assessment framework was established on expert consensus rather than on the research team’s unilateral decisions. The qualitative component used a formalized two-round Delphi procedure rather than interviews or grounded-theory coding. Because the candidate dimensions were literature-derived, the open-ended Round 1 comments were reviewed through focused content analysis and mapped to four assessment functions: starting level (pre-test), final attainment (post-test), amount of change (progress), and the increasing difficulty of further gains (progress difficulty). After wording refinement, Round 2 retained dimensions with a mean importance rating of at least 3.50 and a coefficient of variation no greater than 0.20; the four retained dimensions were then weighted by AHP and converted into the scoring parameters evaluated in Study 2.

After determining the dimensions, this study uses the analytic hierarchy process (AHP) to calculate the weights of the dimensions. AHP is a multi-criteria decision-making method proposed by Saaty in 1980. By constructing a three-level or multi-level structure of goal-criteria-solution, complex decision-making problems are decomposed into more comparable sub-problems, and the importance of indicators is quantified through expert pairwise comparison (Liu, 2021).

It is worth noting that the reference standards and calculation methods for each dimension are also essential. The pre-test and post-test scores refer to the National Students’ Physical Health Standard. The progress score table is used to evaluate the degree of improvement. The use of the progress evaluation table is beneficial to students’ physical education learning. The progressive scoring method is used to evaluate the difficulty of improvement for students, as a significant feature of physical education is that as the level of physical education performance improves, the difficulty of each unit also increases (Holmbeck and Devine, 2009). When formulating evaluation standards, the difficulty of improving performance must be taken into account. The cumulative scoring method is a calculation method that fully considers the difficulty of improving scores.

Finally, for the collection of standing long jump results, the intelligent standing long jump tester (Wang et al., 2024) was selected. One week before the test, the staff participating in the test gathered for pre-testing and training. A unit sample size was randomly selected for the test process drill, allowing the staff to become familiar with the test process and ensuring unified evaluation standards for the results. During the test, each group selected a recorder to record the test results of the participants and inform them of the matters that needed attention during the long jump process at the beginning of the test. The other two test members were responsible for checking whether the students participating in the test had any violations and for broadcasting the results. Each group had to rehearse the entire process and become familiar with it to avoid problems during the formal test. Participants in the formal test completed two standing long jump assessments, one conducted before and one after the semester, with all data fully recorded. Participants who participated in both the first and second-standing long jump tests were selected as valid scores. To maintain anonymity during data collection, participants were required to draw a corresponding number before the first test and use this number when recording their standing long jump results for both the pre- and post-semester trials. The university ethics committee approved this study. The performance test of this study was conducted and collected in public physical education classes. All participants provided signed informed consent.

#### 2.1.3 Statistical analysis

This study first conducted a statistical analysis of the experts’ scores (on a 5-point Likert scale) for each alternative dimension based on the recommendations of Chen et al. (2020). The mean score (Mean), standard deviation (SD), and coefficient of variation (CV = SD/Mean) were computed for each dimension. If the mean score is ≥3.50 and the coefficient of variation (CV) is ≤0.20, the dimension is considered to have both high importance and good consistency. Dimensions that do not meet the above conditions are eliminated to determine the final selection of dimensions. After the dimensions are confirmed, this study uses the Analytic Hierarchy Process (AHP) to assign weights to indicators at the same level. Experts were invited to train on the Saaty 1–5 scaling method, as described by Zhang et al. (2022), and then the indicators at the same level were compared pairwise. According to the paired comparison results of the experts, judgment matrices were constructed for each level. The maximum eigenvalue and corresponding eigenvector of the judgment matrix were solved by the eigenvector method, and the consistency index (CI) and consistency ratio (CR) tests were performed. If CR < 0.10, the matrix was considered to have acceptable consistency; otherwise, the experts were asked to re-correct their scores. Finally, the arithmetic mean of the judgment results of all experts was calculated to obtain the final weights for each dimension.

SPSS.26 data statistical software was used to test the normality of the data, and the VLOOKUP and IF functions were used to match the collected data using Excel office software. Finally, SPSS was used to calculate the results, and the mean score (Mean), standard deviation (SD) and difference standard deviation (SDd) required by the mathematical methods of each dimension were calculated (Tables 1, 2).

**Table 1:** Study 1 demographics characteristics of participants.

Demographics	Categories	Male		Female	
		Number	Percentage (%)	Number	Percentage (%)
Age	<18	186	12.6	454	17.3
	18	352	23.8	660	25.1
	19	581	39.3	860	32.7
	20	306	20.7	510	19.4
	>20	68	4.6	144	5.5
Educational level	Undergraduate Year 1	699	47.3	1,293	49.2
	Undergraduate Year 2	779	52.7	1,336	50.8
Nation	Han ethnic group	810	54.8	1,307	49.7
	Mongolian	102	6.9	168	6.4
	Hui	73	4.9	139	5.3
	Tujia	63	4.3	118	4.5
	Tibetan	57	3.8	103	3.9
	Zhuang	44	3.0	58	2.2
	Manchu	43	2.9	87	3.3
	Uighur	40	2.7	92	3.8
	Other ethnic groups	247	16.7	550	20.9
Total		1,479	100.0	2,630	100.0

**Table 2:** Delphi questionnaire expert survey results.

Dimension (A)	*N*	Mean	SD	CV
Pre-test score (A1)	15	4.000	0.756	0.189
Post-test score (A2)	15	4.600	0.507	0.110
Progress (A3)	15	4.400	0.737	0.167
Progressive Difficulty (A4)	15	4.400	0.633	0.144

#### 2.1.4 Results

Fifteen experts in physical education measurement and evaluation were invited to review the selection of dimensions. Using a 5-point Likert scale, the evaluation experts selected four dimensions. The statistical results showed that the “post-test score” had the highest average score (4.60), the minor standard deviation (0.51), and CV = 0.110, indicating that the experts had the highest recognition of the post-test score dimension and excellent consistency; “progression” and “progression difficulty” were tied for the second highest (both 4.40), of which “progression” CV = 0.144 and “progression difficulty” CV = 0.167, both showing good consistency; “pre-test score” had an average score of 4.00 and CV = 0.189, which was slightly lower than other dimensions, but still in the highly agreed range and with reasonable consistency (0.189 < 0.20). Overall, the CV of each dimension was less than 0.20, and the consistency of expert scoring was good (Vaz et al., 2017), and each dimension could be included in the indicator system.

After the dimensions were determined, the analytic hierarchy process (AHP) was used to compare the indicators at the same level in pairs, using Saaty (2008) 1–5 scale. A judgment matrix was then constructed for each level.

The consistency test is performed, and the formula is


$$\text{λmax}=\frac{1}{n}\sum_{i=1}^{n}\frac{\sum_{i=1}^{n}\text{aijwj}}{\mathrm{wj}}$$

$$\mathrm{CI}=\frac{\lambda \max-n}{n-1}$$

$$\mathrm{CR}=\frac{\mathrm{CI}}{\mathrm{RI}}$$

The results show that when *n* = 4, λmax = 4.0436, RI = 0.90 (Saaty 1980 standard RI table; Brunelli et al., 2013), CI = 0.0145, CR = 0.0161 < 0.10. The consistency test is passed. Therefore, the weight set of the leading indicators is *W* = (0.08, 0.52, 0.20, 0.19) (see Table 3).

**Table 3:** Dimension and weight.

Dimension (A)	Weight
Pre-test score (A1)	0.080
Post-test score (A2)	0.520
Progress (A3)	0.201
Progressive Difficulty (A4)	0.199

The pre-test and post-test scores can be found in the National Students’ Physical Health Standard (Appendix Table A1). Since some scores in the standing long jump evaluation standard table are too vague, in order to make the scores more accurate and reduce errors, the standing long jump evaluation standard table is subdivided (Appendix Tables A2, A3 and see Table 4). For example, the previous long jump score was 272 cm, and the score was 95, but after subdivision, the accurate score was 99. For complete tables, see Appendix Tables A1–A3.

**Table 4:** Comparison table of standing long jump results before and after improvement (partial).

Before		After	
Score	Performance(CM)	Score	Performance(CM)
100	273	100	273
95	268	99	272
		98	271
		97	270
		96	269
		95	268

Calculation of the Improvement Range: This study employed the evaluation method for the improvement range. The *T*-score method was used to measure the student’s initial scores. The calculation formula for the scores was *T* = 50 + 10Z. When calculating the *Z* score,


$$Z=\frac{\left(X-\bar{X}\right)}{S}$$

(X actual score, average score, S standard deviation).

The improvement range was calculated by determining the average score of males and females, as well as the standard deviation (S).


$$T=50+10\frac{\left(X-\bar{X}\right)}{S}$$

( $\text{Male}:\bar{X}$ =58, S = 23; Female: $\bar{X}$ =62, S = 15).

The improvement score is calculated as the difference between the post-test and pre-test scores and is assessed using the progress evaluation table (Appendix Table A4). For example, the pre-test score is 80 points, and the post-test score is 90 points. 90–80 = 10 points is the improvement score. According to the progress evaluation table, the improvement score of 10 points is checked horizontally from left to right in the 80 ~ 84 column. The improvement score is 90 points. However, the progress evaluation table uses two as the interval unit. To ensure the accuracy of the improvement score, the improvement evaluation is refined to use one as the unit of interval (Appendix Tables A5–A7). For example, the pre-test score is 80 points, and the post-test score is 89 points. 89–80 = 9 points are the improvement score. According to the progress evaluation table, the improvement score of 9 points is checked horizontally from left to right in the 80 ~ 84 column. There is no 9 in the query table; only 8 and 10 are present. After subdivision, the exact score corresponding to 9 is 97.5 (Table 5).

**Table 5:** Comparison of the progress evaluation form before and after improvement (Part).

Before							After											
Score	75	80	85	90	95	100	Score	75	77.5	80	82.5	85	87.5	90	92.5	95	97.5	100
95–99	−6	-4	-2	0	2	4	95–99	-6	−5	−4	−3	−2	−1	0	1	2	3	4
90–94	−4	−2	0	2	4	6	90–94	−4	−3	−2	−1	0	1	2	3	4	5	6
85–89	−2	0	2	4	6	8	85–89	−2	−1	0	1	2	3	4	5	6	7	8
80–84	0	2	4	6	8	10	80–84	0	1	2	3	4	5	6	7	8	9	10

Considering the increasing marginal difficulty of improving physical skills (Holmbeck and Devine, 2009), this study used a cumulative scoring approach to rate improvement difficulty. When formulating evaluation criteria, the difficulty of improving performance must be taken into account. Cumulative scoring is a technique that entirely takes into account the difficulty of improving scores (Mohzana et al., 2024). Its calculation formula. Here, y represents the cumulative score, k is the coefficient, D is the variable, and Z is the constant. The characteristic is that as the value of variable D increases, the cumulative score y obtained by increasing the value by one unit will also increase. First, the coefficient k and the constant Z must be determined. The value of D can be obtained by consulting the corresponding table of variables X, U and D (Table 6). The commonly used scoring range is the mean score plus or minus three standard deviations, which accounts for 99.73% of the total frequency as the full score and starting score. Assuming a maximum score of 100 points and a minimum score of 0 points. The scoring range is *μ* ± 3S. If the full score is μ + 3S, then the D value table shows D100 = 8. If the minimum score is μ- 3S, then the D value table shows D0 = 2. $100=K\cdot 8^{2}-Z$ and $0=K\cdot 2^{2}-Z$ . K = 1.67; Z = 6.67. Substituting K and Z values into:y = 1.67D2 − 6.67. The calculation formula for the D value corresponding to the standing long jump is: $D=5+\frac{X-\bar{X}}{S}$ (X is the post-test score - pre-test score is the average improvement score, and S is the standard deviation of the average improvement score). Average progress score calculation formula:


$$\bar{x}=\bar{d}=\frac{1}{n}\sum_{i=1}^{n}d_{i}$$

**Table 6:** Mapping of X, U, and D variables.

Primitive variable X	$\bar{X}$	$\bar{X}$	$\bar{X}$	$\bar{X}$	$\bar{X}$	$\bar{X}$	$\bar{X}$	$\bar{X}$	$\bar{X}$	$\bar{X}$	$\bar{X}$
	−	−	−	−	−		+	+	+	+	+
	5S	4S	3S	2S	S		S	2S	3S	4S	5S
Standard variable U	−5	−4	−3	−2	−1	0	1	2	3	4	5
Variable D	0	1	2	3	4	5	6	7	8	9	10

The formula for calculating individual difference is:


$$X=d_{i}=X_{\text{post},i}-X_{\mathrm{pre},i}$$

The formula for calculating the standard deviation of the average improvement score is:


$$S=S_{d}=\sqrt{\frac{1}{n-1}\sum_{i=1}^{n}{\left(d_{i}-\bar{d}\right)}^{2}}$$

Progressive Difficulty Formula:


$$y=1.67*{\left(5+\frac{X-\bar{X}}{S}\right)}^{2}-6.67$$

( $\text{Male}:\bar{X}$ =5.35, S = 11.41; $\text{Female}:\bar{X}$ =8.30; S = 10.85)

The final task-specific standard referenced each student’s baseline and growth trajectory while retaining end-point attainment. It combined baseline performance, end-point attainment, improvement magnitude, and improvement difficulty in a weighted composite score. This standard is intended only for standing long jump performance in the sampled university context. The transferable contribution lies in the development workflow—expert refinement, local calibration, transparent weighting, and classroom evaluation—rather than in treating the resulting weights or cut scores as universal (Table 7).

**Table 7:** Study 2 demographics characteristics of participants.

Demographics	Categories	Male		Female	
		Number	Percentage (%)	Number	Percentage (%)
Age	<18	11	7.9	19	14.2
	18	42	30.2	36	26.9
	19	57	41.0	37	27.6
	20	25	18.0	33	24.6
	>20	4	2.9	9	6.7
Educational level	Undergraduate Year 1	69	49.6	66	49.3
	Undergraduate Year 2	70	50.4	68	50.7
Nation	Han ethnic group	79	56.7	59	43.9
	Mongolian	7	5.0	11	8.2
	Hui	4	2.9	5	3.7
	Tujia	6	4.3	7	5.2
	Tibetan	7	5.0	5	3.9
	Zhuang	4	3.0	2	1.5
	Manchu	2	1.4	6	4.5
	Uighur	3	2.2	1	0.7
	Other ethnic groups	27	19.4	38	28.4
Total		139	100.0	134	100.0

### 2.2 Study 2

Study 2 evaluated whether the standing long jump assessment standard developed in Study 1 functioned as a motivation-supportive classroom intervention. Classes assigned to the intervention group used the four-dimension progress-based standard, whereas control classes continued to use the conventional absolute-score approach. The comparison therefore concerns assessment of one fitness task and should not be interpreted as a comparison of comprehensive physical education curricula.

#### 2.2.1 Participants

This study targeted students enrolled in public physical education courses at the university level. This choice was made for three main reasons. First, the goals and instructional structure of such courses are relatively standardized, which facilitates the implementation of assessment reform in authentic classroom settings. Second, university students are more likely to understand and make use of progress-based feedback. Third, students in these courses typically show substantial baseline differences in physical fitness, which provides a suitable context for testing how a progress-referenced assessment mechanism influences motivation and performance. The study was conducted at an ethnic-minority university, where the multi-ethnic and diverse student composition may shape students’ sensitivity to assessment fairness and feedback support. Accordingly, the findings should be interpreted as evidence of effectiveness within this specific institutional context, and further validation is needed before generalizing the results to other educational stages or types of institutions. The experiment to evaluate the multidimensional evaluation criteria was conducted at Minzu University of China. The participants were first- and second-year undergraduates who were typically enrolled in public physical education classes. Group assignment was determined through class-level cluster randomization. It should be noted that this study adopted a two-stage “development–evaluation” design, and the difference in sample sizes across the two stages reflects distinct research aims and statistical requirements. Study 1 aimed to develop the multidimensional progress-based assessment system and calibrate its parameters. A relatively large sample was therefore required to enhance the stability of the dimensional structure and scoring parameters. Study 2 focused on evaluating the practical effectiveness of the assessment system as a classroom intervention tool. The sample size for Study 2 was determined *a priori* using G*Power to ensure adequate statistical power for detecting the intervention effect (Ringle et al., 2020). With a two-tailed significance level of *α* = 0.05 and power of 1 − *β* = 0.80, and based on the expected effect size reported in prior physical education intervention studies, the minimum required sample size for Study 2 was estimated as N = 256 (128 students per group). Accordingly, the second-stage sample size represents a reasonable choice grounded in power analysis and classroom implementation feasibility, and it was not intended to match the sample size of Study 1.

A total of 307 students were assessed for eligibility. Fifteen students were excluded for the following reasons: not meeting the inclusion criteria (*n* = 3), refusal to participate (*n* = 10), injury (*n* = 1), and other reasons (*n* = 1). The remaining 292 students were allocated to groups: 147 in the intervention group (73 males, 74 females) and 145 in the control group (72 males, 73 females). The final sample included in the primary DID analyses was 273 students: 141 in the intervention group (72 males, 69 females) and 132 in the control group (67 males, 65 females). An additional 19 students were not included in the complete-case analyses due to missing pre- and/or post-test data (6 in the intervention group and 13 in the control group). Missingness was mainly attributable to absences during the intervention period, illness, and incomplete questionnaire responses.

#### 2.2.2 Method

This study was divided into an intervention group and a control group. The intervention group was evaluated according to multidimensional criteria, while the control group was evaluated according to the original criteria. To enhance implementation fidelity and to clarify the intended transparency contrast between the two groups, this study standardized the communication and implementation procedures for the assessment approach. Before the intervention, teachers in the intervention group received unified training, and each teacher explained the multidimensional assessment rules to students in the first class session. The control group continued with conventional assessment practices and was informed only of the basic assessment requirements and the composition of the final grade, without introducing the scoring rules centered on the multidimensional assessment standard. To minimize implementation bias, the two groups followed the same instructional content, instructional time allocation, and testing procedures. The experimental period lasted 16 weeks (September to December 2023), and the experimental school selected was Minzu University of China. Minzu University of China is a comprehensive university in China, and the school has diverse cultural, ethnic and physical education foundations, which helps to improve the external validity of the research conclusions and the feasibility of promotion. This study employed the Difference-in-Differences (DID) model to evaluate the research results, calculating the difference in scores between the IG and the CG before and after the intervention. Subsequently, an independent samples t-test was performed to assess the difference between the two groups and determine the net effect of the evaluation criteria (Fredriksson and Oliveira, 2019). The DID model is based on the ordinary least squares (OLS) regression model. In statistics and econometrics, Ordinary Least Squares (OLS) regression is a classic method for estimating the parameters of a linear regression model by minimizing the sum of squares of residuals between the observed and predicted values. The straight line constructed is the “best-fit line” (James et al., 2023). OLS modifies the model’s intercept and slope coefficient to minimize the sum of squared distances from all sample points to the regression line, thus achieving the best linear representation of the relationship between the dependent and independent variables (Wooditch et al., 2021).

Before the experiment officially begins, all participating teachers will be trained together to explain the test process and scoring criteria in a unified manner. A unit of samples will be randomly selected for full-process drills to ensure that each teacher is familiar with the key points of the operation. Each group will assign a recorder to record the results of each participant and remind them of precautions before the test begins; another one to two members will be responsible for supervising the test process, checking whether there are any violations, and reporting and correcting them promptly; before the full-process test, the staff will lead the participants to fully warm up and activate the joints and muscles, which can not only avoid physical education injuries but also ensure that students can perform at their proper level. Before and after the semester, all participants were given a standing long jump test. Samples with complete scores for both tests were collected as valid data. If both feet or any toe touched the line or crossed the line during take-off, the score would be invalidated. If the participant leaned back or his feet landed on the mat and fell back, the distance of the landing point closest to the take-off point would be recorded. Any action that did not meet the standard was recorded as an invalid score.

For measuring learning motivation, the “Work Preference Scale for Motivation Measurement” was selected to assess intrinsic and extrinsic motivation orientations (Amabile et al., 1994). To ensure linguistic and contextual equivalence, the scale was adapted into Chinese following a standard translation procedure. Two bilingual researchers independently conducted forward translation. Discrepancies were resolved through discussion, and a third bilingual expert completed back translation. An expert panel with experience in physical education and educational measurement reviewed the translated items to ensure clarity and applicability to the university physical education context. A small-scale pilot test was conducted to confirm item comprehensibility. Minor wording refinements were made to better fit the physical education learning setting, without altering the original meaning. The final Chinese version retained the original two-factor structure (intrinsic motivation and extrinsic motivation), comprising 30 items rated on a five-point Likert scale (1 = strongly disagree, 5 = strongly agree). Structural validity was examined using confirmatory factor analysis (CFA). Internal consistency was high for both intrinsic motivation and extrinsic motivation (Cronbach’s *α* = 0.835–0.977) (Cronbach, 1951). It should be noted that when a scale includes many items and items are highly homogeneous, α values may be very high. However, α alone does not demonstrate unidimensionality or indicate data manipulation (Taber, 2018). To address potential redundancy, we further examined inter-item correlations and item–total correlations and did not identify duplicated items. To test whether the net intervention effect varied by gender, we conducted a formal gender-difference test within the DID framework. Specifically, we compared the Treat × Post (β3) estimates between male and female subsamples and reported the difference estimate captured by Treat × Post × Gender, together with its confidence interval.

To ensure anonymity, participants randomly drew an identification code before the first measurement. Only this code was used to record both standing long jump test results and both motivation questionnaire responses, and no personally identifiable information was collected. This study received approval from the university ethics committee. Both performance tests and questionnaires were administered and collected during public physical education classes, and written informed consent was obtained from all participants.

#### 2.2.3 Statistical analysis

Normality tests were first conducted on the two motivation subscales (extrinsic motivation and intrinsic motivation; 15 items each; all rated on a 5-point Likert scale from 1 = “strongly disagree” to 5 = “strongly agree”) using the pre-test data. After the distributions met the normality assumption, we proceeded to assess the parallel trends assumption. We then examined common method bias (CMB) (Podsakoff et al., 2003). As a supplementary diagnostic, we applied the single-factor CFA comparison approach by specifying a one-factor model in which all measurement items loaded onto a single latent construct and comparing its fit with that of the theorized multi-factor measurement model. The results indicated that the theorized multi-factor model fit the data well (χ^2^ = 658.412, df = 404, χ^2^/df = 1.630; RMSEA = 0.048, 90% CI [0.041, 0.055]; SRMR = 0.032; CFI = 0.973; TLI = 0.971; NFI = 0.933; GFI = 0.864; AGFI = 0.843; PGFI = 0.750; *N* = 273). In contrast, the one-factor model showed a substantially poorer fit (χ^2^ = 3593.625, df = 405, χ^2^/df = 8.873; RMSEA = 0.170, 90% CI [0.165, 0.175]; SRMR = 0.155; CFI = 0.662; TLI = 0.637; NFI = 0.636; GFI = 0.213; AGFI = 0.096; PGFI = 0.185; *N* = 273). These findings suggest that a single method factor is unlikely to account for the covariance structure among the items; therefore, CMB is unlikely to have materially biased the conclusions of this study. The two subscales of the motivation questionnaire (external motivation and internal motivation, 15 questions each, both using a 5-point Likert scale) were tested for normality in the pre-test, and a parallel trend test was performed after the results met normality. Subsequently, the difference between the pre-test mean and the post-test mean was calculated for each subject’s total score on the motivation questionnaire and the overall mean score. The specific calculation formula is:


$$\;d_{i}=X_{\text{post},i}-X_{\mathrm{pre},i}$$

$$S_{d}=\sqrt{\frac{1}{n-1}\sum_{i=1}^{n}{\left(d_{i}-\bar{d}\right)}^{2}}$$

**Figure 1:**
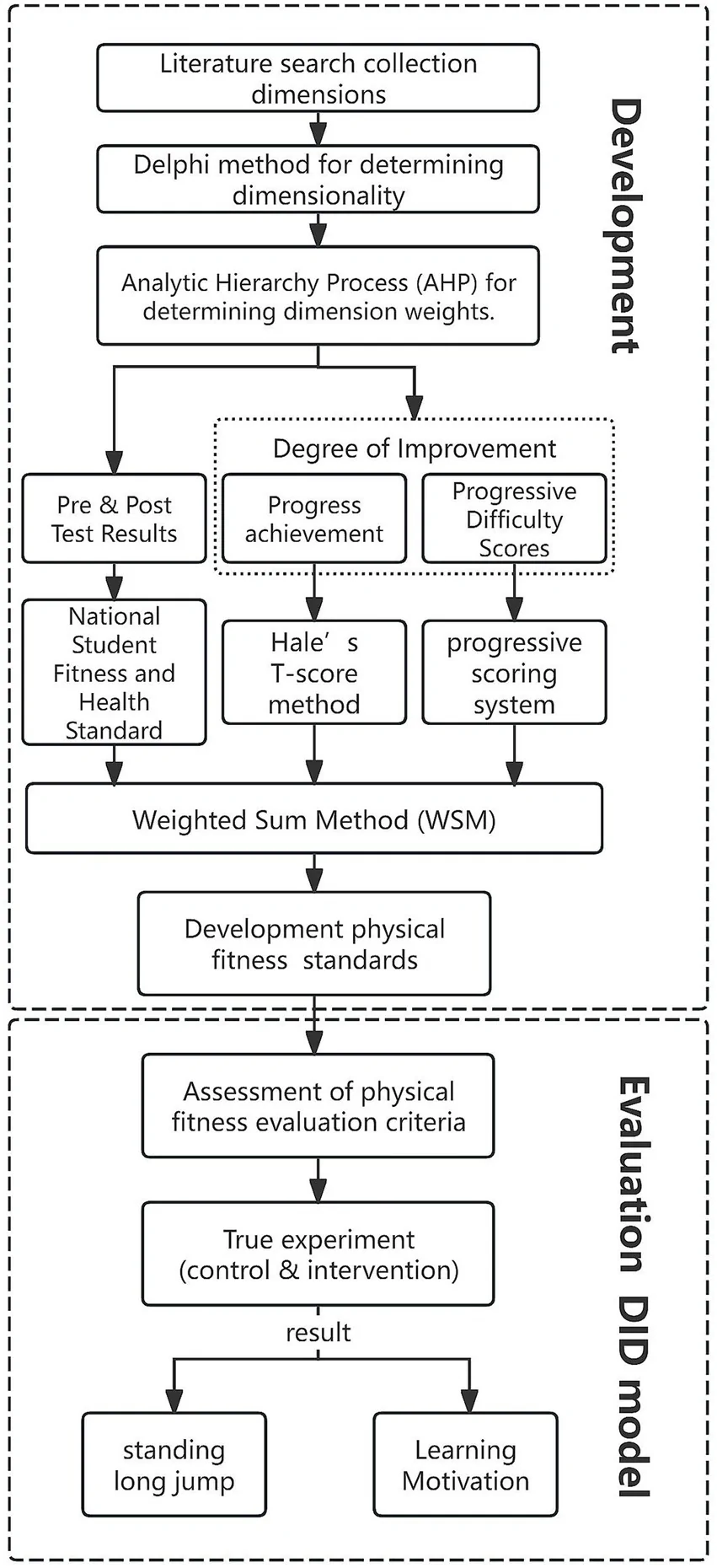
Study design flow chart.

**Figure 2:**
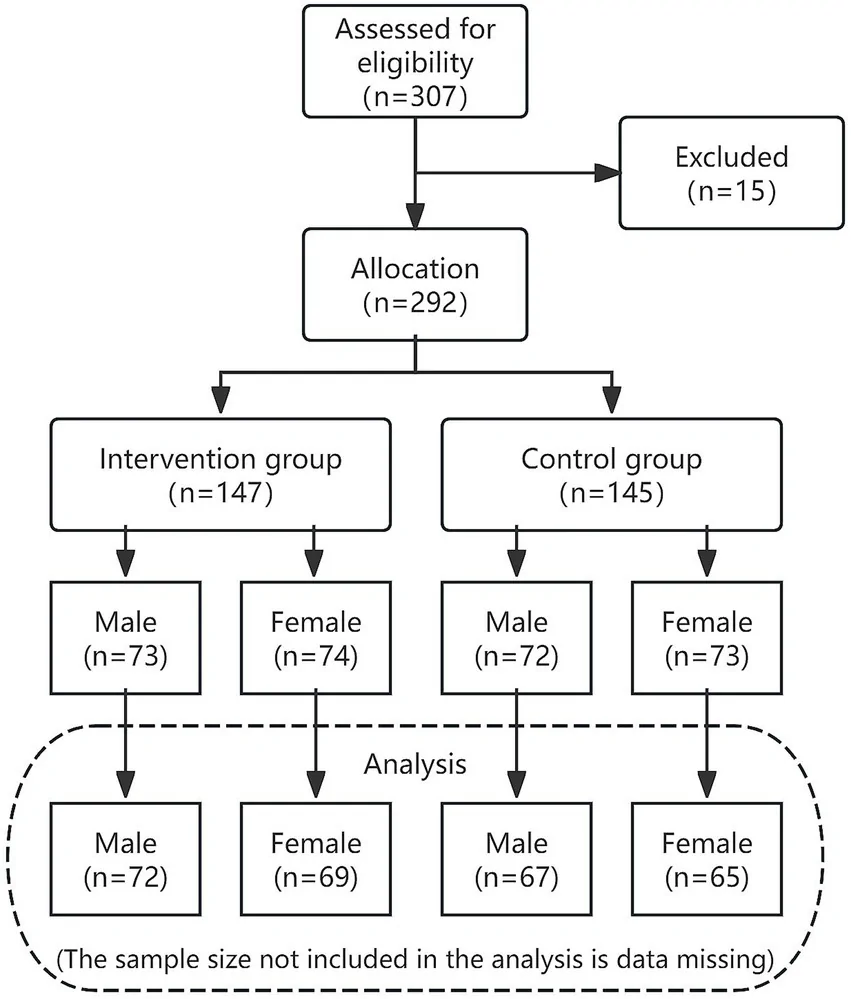
Study 2 participant allocation diagram.

Based on the mean difference, the difference is tested for normality. When the difference result meets the normality test, the DID framework is used to estimate the intervention effect of the IG relative to the CG. This is equivalent to an independent sample *t*-test (two-sided, *α* = 0.05) on the mean difference between the two groups, with a Levene correction applied for heterogeneity of the differences. Finally, the net impact results of the multi-dimensional evaluation criteria are obtained to determine whether they are significant and whether the hypothesis is established (Figures 1, 2).

#### 2.2.4 Results

At baseline, skewness ranged from −0.636 to 0.990 and kurtosis ranged from −1.335 to 1.089 for the control and intervention groups among both male and female students (Appendix Table A9). All values fell within the commonly used threshold of [−2, 2], indicating that the pretest data were approximately normally distributed. This distributional pattern satisfies the parametric prerequisite for subsequent parallel-trend testing (Hatem et al., 2022). Building on this assessment, we further examined pre-intervention baseline differences between groups on key indicators and reported standardized mean differences (SMDs). As shown in Table 8, the intervention and control groups exhibited good baseline comparability, which supports the DID identifying assumption. Regression estimates were obtained using class-level cluster-robust standard errors.

**Table 8:** Parallel trend test results for males and females before intervention.

Gender	Index	Mean	Mean difference	Std. error difference	*P* (Levene’s test)	*t*	*p*	SMD
Male	Internal motivation	3.088	0.022	0.151	0.000	0.145	0.885	0.025
	External motivation	3.095	0.007	0.152	0.039	0.043	0.966	0.008
	Standing long jump results	221.700	−1.187	3.440	0.900	−0.345	0.730	0.058
Female	Internal motivation	3.078	−0.006	0.162	0.640	−0.038	0.970	0.006
	External motivation	3.222	−0.007	0.175	0.404	−0.042	0.966	0.007
	Standing long jump results	159.510	0.957	2.674	0.402	0.358	0.721	0.062

The parallel-trend tests indicated that, for both male and female students, pre-intervention differences between the intervention and control groups were not statistically significant across all key indicators, thereby supporting the parallel trends assumption. To ensure an appropriate variance specification for group comparisons, we selected the corresponding t-test output based on Levene’s test of homogeneity of variances: when p(Levene’s test) < 0.05, we reported results under “equal variances not assumed,” whereas when p(Levene’s test) > 0.05, we reported results under “equal variances assumed” (Asrial et al., 2022). For the male subsample, the pre-intervention Levene’s test was significant for intrinsic motivation (*p* = 0.000), yet the between-group difference was not significant (*p* = 0.885 > 0.05). A similar pattern was observed for extrinsic motivation (Levene’s *p* = 0.039; between-group *p* = 0.966 > 0.05). For standing long jump performance, Levene’s test was not significant (Levene’s *p* = 0.900), and the between-group difference remained non-significant (*p* = 0.730 > 0.05). The female subsample showed consistent results: intrinsic motivation (Levene’s *p* = 0.640; between-group *p* = 0.970 > 0.05), extrinsic motivation (Levene’s *p* = 0.404; between-group p = 0.966 > 0.05), and standing long jump performance (Levene’s *p* = 0.402; between-group *p* = 0.721 > 0.05) all exhibited non-significant differences. Overall, all between-group *p* values were clearly greater than 0.05, indicating no systematic pre-intervention differences between the intervention and control groups on the focal indicators. Baseline imbalance was also minimal (|SMD| ≤ 0.062), suggesting strong baseline comparability. These results support the core prerequisite for DID estimation, namely that the two groups exhibited similar outcome trajectories prior to the intervention (Egami and Yamauchi, 2023).

Building on this evidence, we further report descriptive statistics for male and female students before and after the intervention to provide an intuitive picture of changes over time. Table 9 presents the pretest and posttest means (M) and standard deviations (SD) for intrinsic motivation, extrinsic motivation, and standing long jump performance in the intervention and control groups. Overall, the pattern was consistent across genders: compared with the control group, the intervention group showed larger improvements in both types of motivation and in standing long jump performance. Specifically, intrinsic motivation in the intervention group increased by approximately 1.10–1.13 points and extrinsic motivation increased by approximately 1.05–1.08 points, whereas the control group showed smaller gains in both types of motivation (approximately 0.18–0.26 points). For standing long jump performance, the intervention group improved by approximately 20–23 cm on average, whereas the control group improved by approximately 5–8 cm. This descriptive pattern provides intuitive support for the subsequent DID estimation of the net intervention effect.

**Table 9:** The mean and standard deviation of each index before and after the male and female intervention groups.

Group/Gender	*N*	Index	Time node	*M*	SD
IG (MALE)	72	Internal motivation	Pre	3.066	0.731
			Post	4.169	0.399
		External Motivation	Pre	3.088	0.810
			Post	4.133	0.535
		Standing long jump results	Pre	222.875	20.385
			Post	246.347	17.673
CG (MALE)	67	Internal motivation	Pre	3.088	1.011
			Post	3.272	0.914
		External motivation	Pre	3.095	0.963
			Post	3.337	0.870
		Standing long jump results	Pre	221.701	19.822
			Post	226.910	18.225
IG (FEMALE)	69	Internal motivation	Pre	3.084	0.905
			Post	4.218	0.387
		External motivation	Pre	3.229	1.038
			Post	4.306	0.421
		Standing long jump results	Pre	158.551	14.792
			Post	178.493	14.916
CG (FEMALE)	65	Internal motivation	Pre	3.078	0.949
			Post	3.334	0.898
		External motivation	Pre	3.222	0.973
			Post	3.467	0.790
		Standing long jump results	Pre	159.508	15.929
			Post	167.400	15.126

After presenting the overall pre–post changes in the focal outcomes, we further examined the distributional assumptions required for parametric tests. Based on the pre–post change scores for the three indicators, skewness ranged from −0.259 to 1.358 and kurtosis ranged from −1.147 to 1.345 (Appendix Table A10). All values fell within the commonly used [−2, 2] threshold, indicating that the change-score distributions were approximately normal (Hatem et al., 2022). This result supports the subsequent use of parametric methods, including t tests and DID regressions.

Given that the normality assumption was satisfied, we then compared the magnitude of within-group changes between the intervention and control groups. Table 10 summarizes the means (M), standard deviations (SD), standard errors (SE Mean), *p* values, and confidence intervals (CIs) for both within-group change scores and between-group differences in change scores across the three indicators. For intrinsic and extrinsic motivation, Levene’s tests were significant (*p* < 0.001), indicating heteroscedasticity. We therefore used Welch’s t test (i.e., the unequal-variances *t*-test) for variance correction (Kent State University Libraries, 2017). Prior work suggests that Welch’s t test provides more robust control of Type I error when group variances differ substantially or sample sizes are unequal, while yielding results that are nearly identical to the classic *t*-test when variances are in fact equal (West, 2021).

**Table 10:** M, SD, SE Mean, P, CI of the differences within and between the IG and the CG.

Gender	Index	Group	*N*	Mean	SD	SE Mean	*P* (Levene’s Test)	Mean	*t*	*p*	BH-FDRq	SE (SE Mean)	95% CI	
Male	Internal motivation	CG	67	0.184	0.166	0.020	0.000	−0.919	−15.542	0.000	0.001	0.059	−1.036	−0.802
		IG	72	1.103	0.457	0.054								
	External motivation	CG	67	0.243	0.216	0.026	0.000	−0.803	−13.765	0.000	0.001	0.058	−0.918	−0.687
		IG	72	1.045	0.429	0.051								
	Standing long jump results	CG	67	5.210	6.428	0.785	0.405	−18.263	−15.223	0.000	0.001	1.200	−20.636	−15.891
		IG	72	23.470	7.614	0.897								
Female	Internal motivation	CG	65	0.256	0.219	0.027	0.000	−0.878	−11.099	0.000	0.001	0.079	−1.034	−0.721
		IG	69	1.134	0.601	0.072								
	External Motivation	CG	65	0.245	0.245	0.030	0.000	−0.832	−8.662	0.000	0.001	0.096	−1.022	−0.642
		IG	69	1.077	0.737	0.089								
	Standing long jump results	CG	65	7.890	6.450	0.800	0.057	−12.050	−9.303	0.029	0.029	1.295	−14.613	−9.486
		IG	69	19.940	8.463	1.019								

Mean is the mean of the posttest–pretest differences; SD is the standard deviation of the posttest - pretest differences; SE is the standard error of the posttest–pretest differences; Mean is the mean of the CG difference - the mean of the IG difference.

For intrinsic motivation, the male control group showed a within-group change of *M* = 0.184 (SD = 0.166), whereas the male intervention group showed a change of *M* = 1.103 (SD = 0.457). The improvement in the intervention group was approximately five times that of the control group, and the change scores were more dispersed. The between-group difference was statistically significant (*p* < 0.001), indicating that the intervention significantly increased male students’ intrinsic motivation. A parallel pattern was observed for female students: the control group change was *M* = 0.256 (SD = 0.219), whereas the intervention group change was *M* = 1.134 (SD = 0.601). The difference was also significant (*p* < 0.001), suggesting a stable intervention effect on female students’ intrinsic motivation.

For extrinsic motivation, the male control group showed a within-group change of *M* = 0.243 (SD = 0.216), whereas the male intervention group showed a change of *M* = 1.045 (SD = 0.429). The Welch-corrected test yielded *p* < 0.001, indicating a significant increase in male students’ extrinsic motivation attributable to the intervention. The female subsample showed a consistent result: the control group change was *M* = 0.245 (SD = 0.245) and the intervention group change was *M* = 1.077 (SD = 0.737), with the difference again significant (*p* < 0.001). This finding further supports the effectiveness of the intervention in enhancing female students’ extrinsic motivation.

For standing long jump performance, the male control group improved by *M* = 5.210 cm (SD = 6.428), whereas the male intervention group improved by *M* = 23.470 cm (SD = 7.614). Levene’s test was non-significant (*p* = 0.405 > 0.05), suggesting that the equal-variance assumption was tenable. The between-group difference was significant (*p* < 0.001), indicating that the intervention produced a substantial improvement in male students’ standing long jump performance. For female students, the control group improved by *M* = 7.890 cm (SD = 6.450) and the intervention group improved by *M* = 19.940 cm (SD = 8.463). Although Levene’s test was marginal (*p* = 0.057 > 0.05), we relied on the variance-corrected test results given its proximity to the conventional threshold. The between-group difference remained significant (*p* < 0.01), suggesting that the intervention also significantly improved female students’ standing long jump performance.

To control the false discovery risk arising from multiple comparisons, we applied the Benjamini–Hochberg false discovery rate (BH–FDR) correction. The correction was implemented within each gender across the three primary outcome indicators. After adjustment, all effects remained statistically significant (all *q* < 0.05), indicating that the conclusions were robust to multiple-testing control.

After controlling for baseline levels and common time trends, the DID estimates clearly indicated an incremental intervention effect on intrinsic motivation (Table 11). The interaction term was significantly positive in the male subsample (*β*₃ = 0.86, *p* < 0.001), suggesting that the intervention group achieved additional gains after the intervention relative to the control group. A consistent and robust positive effect was also observed in the female subsample (β₃ = 0.88, *p* < 0.001). The corresponding standardized effect sizes were large (male *d* ≈ 0.98; female *d* ≈ 0.95), indicating that the assessment reform was not only statistically significant but also practically meaningful, with a comparable facilitative effect across genders.

**Table 11:** OLS estimates of the DID specification of internal motivation.

Gender	Coefficient	*B*	SE	*t*	*p*	95% CI		R^2^	DID regression model
Male (*N* = 278)	β_0_	3.094	0.094	32.854	0.000	2.909	3.280	0.259	Y_it_ = 3.094 + (−0.029)Treat_i_ + 0.243Post_t_ + 0.860(Treat_i_ × Post_t_) + ε_it_.
	β_1_	−0.029	0.131	−0.220	0.826	−0.286	0.229		
	β_2_	0.243	0.133	1.823	0.069	−0.019	0.505		
	β_3_	0.860	0.185	4.646	0.000	0.496	1.224		
Female (*N* = 268)	β_0_	3.078	0.102	30.238	0.000	2.877	3.278	0.252	Y_it_ = 3.078 + 0.006Treat_i_ + 0.256Post_t_ + 0.878(Treat_i_ × Post_t_) + ε_it_.
	β_1_	0.006	0.142	0.043	0.965	−0.273	0.285		
	β_2_	0.256	0.144	1.781	0.076	−0.027	0.540		
	β_3_	0.878	0.201	4.376	0.000	0.483	1.273		

**Figure 3:**
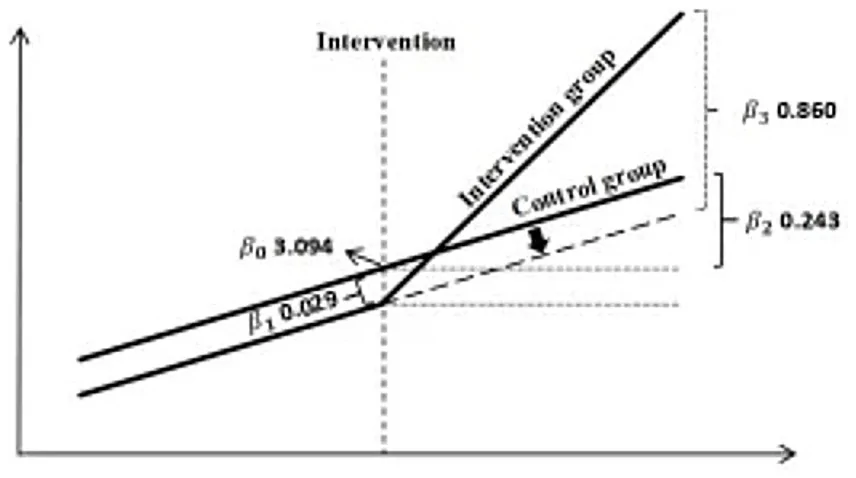
DID model of boys’ internal motivation.

**Figure 4:**
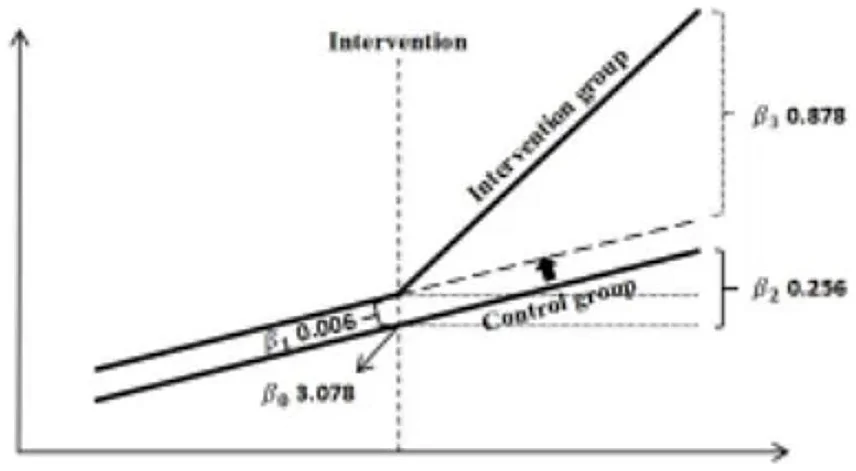
DID model of Girls’ internal motivation.

As depicted in Figures 3, 4, the two groups were highly similar in their baseline levels prior to the intervention, and their pre-intervention trajectories were comparable, as reflected by *β*₁ being close to zero. After implementation, intrinsic motivation increased only slightly in the control group (male: *β*₂ = 0.243; female: β₂ = 0.256), whereas the intervention group showed a much larger gain, with the total increase reaching β₂ + β₃ (male: 1.103; female: 1.134). Moreover, the vertical separation between the two predicted post-intervention trajectories corresponds to the interaction term β₃ (male: 0.860; female: 0.878), which provides intuitive support for the interpretation that the additional gains were primarily attributable to the intervention.

For extrinsic motivation, the DID estimates likewise provided clear evidence of a net intervention effect (Table 12). In the male subsample, the intervention group showed an additional gain of approximately 0.80 relative to the control group (*p* < 0.001). The corresponding net gain in the female subsample was approximately 0.83 and was also highly significant. The effect-size results further reinforce this conclusion: the standardized effect sizes were in the upper-moderate range (male *d* ≈ 0.91; female *d* ≈ 0.83), indicating that the assessment reform had not only statistically significant but also substantively meaningful effects on extrinsic motivation. The effect sizes were relatively similar across genders, suggesting no pronounced gender differentiation in the impact of the reform.

**Table 12:** OLS estimates of the externally motivated DID specification.

Gender	Coefficient	B	SE	*t*	*p*	95% CI		*R* ^2^	DID regression model
Male (*N* = 278)	β_0_	3.095	0.099	31.193	0.000	2.899	3.290	0.224	Y_it_ = 3.095 + (−0.007)Treat_i_ + 0.243Post_t_ + 0.803(Treat_i_ × Post_t_) + ε_it_.
	β_1_	−0.007	0.138	−0.047	0.962	−0.278	0.265		
	β_2_	0.243	0.140	1.731	0.085	−0.033	0.519		
	β_3_	0.803	0.195	4.117	0.000	0.419	1.186		
Female (*N* = 268)	β_0_	3.222	0.105	30.728	0.000	3.015	3.428	0.223	Y_it_ = 3.222 + 0.007Treat_i_ + 0.245Post_t_ + 0.832(Treat_i_ × Post_t_) + ε_it_.
	β_1_	0.007	0.146	0.051	0.960	−0.280	0.295		
	β_2_	0.245	0.148	1.653	0.099	−0.047	0.537		
	β_3_	0.832	0.207	4.027	0.000	0.425	1.239		

**Figure 5:**
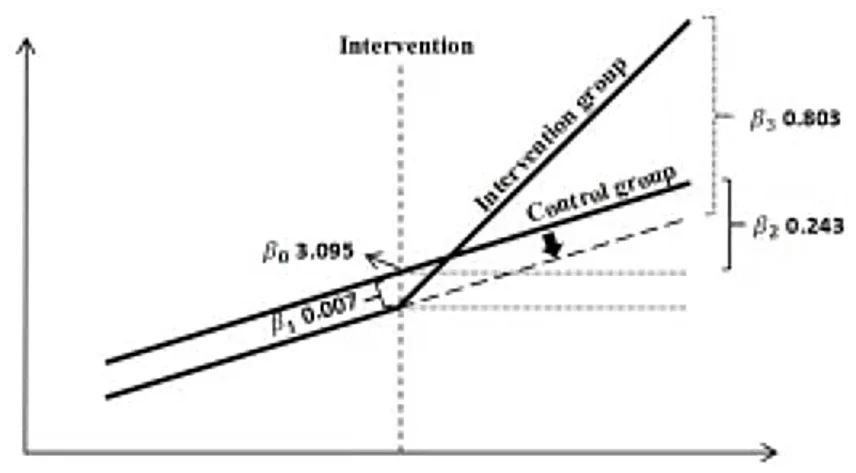
DID model of external motivation for boys.

**Figure 6:**
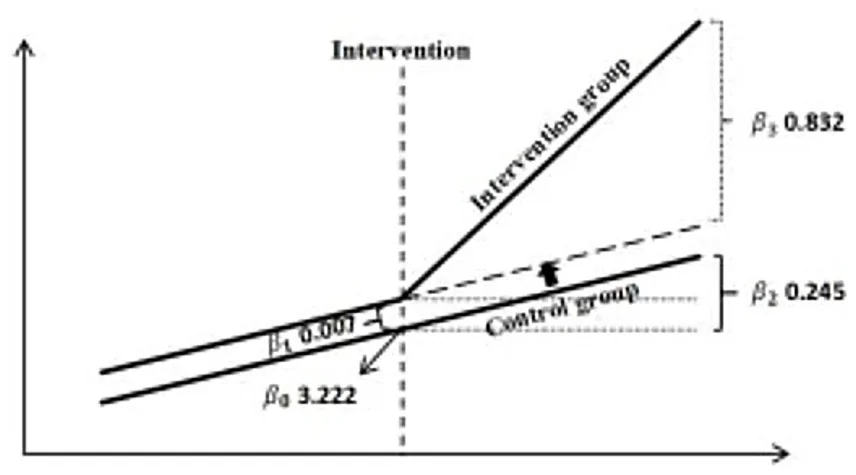
DID model of external motivation for girls.

Consistent with the statistical estimates, Figures 5, 6 show a canonical “similar pre-intervention levels, divergent post-intervention trajectories” pattern for extrinsic motivation. The pre-intervention baseline offset was minimal (*β*₁ close to 0). In the absence of the reform, the control group exhibited only a modest upward shift along the existing trend (male: β₂ = 0.243; female: β₂ = 0.245). In contrast, the intervention group increased to β₂ + β₃ after implementation (male: 1.037; female: 1.077). The post-intervention vertical gap between the two predicted trajectories also aligns with the interaction term β₃ (male: 0.803; female: 0.832), indicating that the additional improvement in extrinsic motivation corresponds closely to the implementation of the assessment reform.

Furthermore, the physical performance results provide additional evidence for the overall utility of the intervention (Table 13). For standing long jump performance, the DID interaction term was approximately 18.26 in the male subsample (*p* < 0.001) and approximately 12.05 in the female subsample (*p* < 0.01), indicating that performance gains in the intervention group exceeded what would be expected from natural trends alone. The corresponding standardized effect sizes were in the moderate-to-large range (male *d* ≈ 0.91; female *d* ≈ 0.78). These results suggest that the assessment reform not only enhanced students’ motivation but also translated into observable improvements in physical performance. The direction of the effects was consistent across genders, with no pronounced gender divergence.

**Table 13:** OLS estimates of the DID norm for standing long jump performance.

Gender	Coefficient	B	SE	*t*	*p*	95% CI		*R* ^2^	DID regression model
Male (*N* = 278)	β_0_	221.701	2.345	94.526	0.000	217.084	226.319	0.218	Y_it_ = 221.701 + 1.174Treat_i_ + 5.209Post_t_ + 18.263(Treat_i_ × Post_t_) + ε_it_.
	β_1_	1.174	3.259	0.360	0.719	−5.242	7.589		
	β_2_	5.209	3.317	1.570	0.117	−1.321	11.739		
	β_3_	18.263	4.609	3.963	0.000	9.190	27.336		
Female (*N* = 268)	β_0_	159.508	1.898	84.043	0.000	155.771	163.245	0.220	Y_it_ = 159.508 + (−0.957)Treat_i_ + 7.892Post_t_ + 12.050(Treat_i_ × Post_t_) + ε_it_.
	β_1_	−0.957	2.645	−0.362	0.718	−6.165	4.251		
	β_2_	7.892	2.684	2.940	0.004	2.607	13.177		
	β_3_	12.050	3.740	3.221	0.001	4.685	19.415		

**Figure 7:**
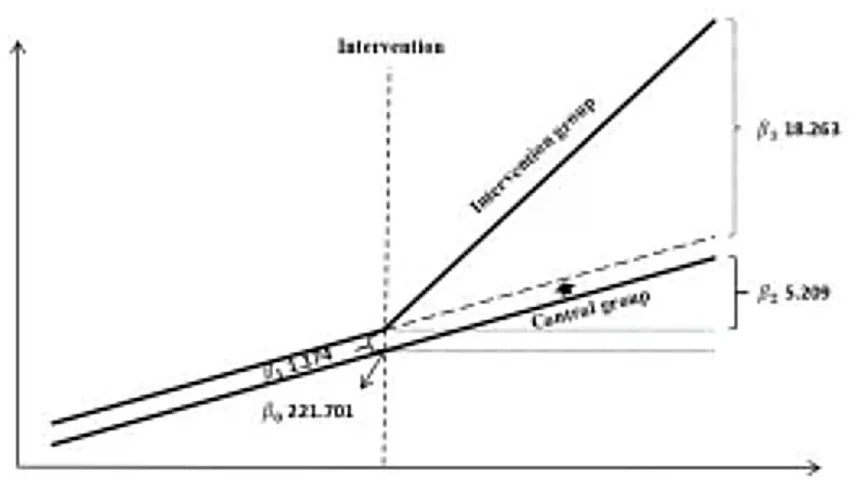
DID model diagram for boys’ standing long jump results.

**Figure 8:**
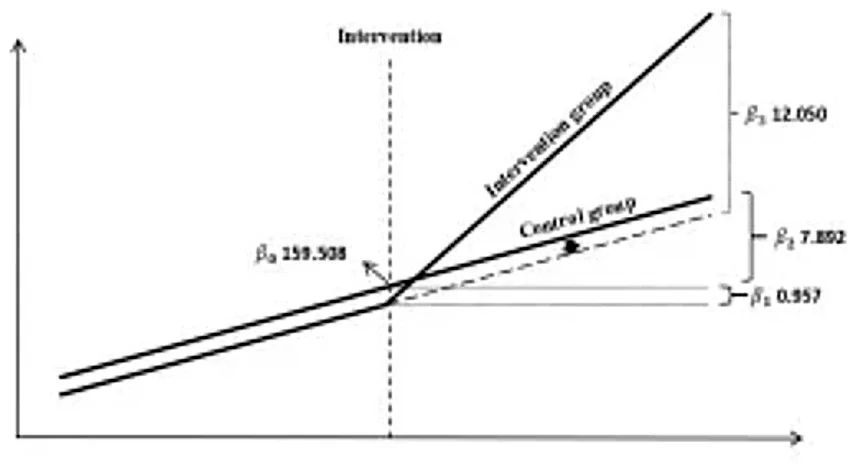
DID model diagram of standing long jump results for girls.

Figures 7, 8 provide a more intuitive interpretation of this pattern. Prior to the intervention, both male and female samples showed comparable starting points and similar upward slopes. After implementation, performance in the control group increased only by β₂ (male: 5.209; female: 7.892), whereas the intervention group moved substantially ahead, with the total gain reaching β₂ + β₃ (male: 23.472; female: 19.942). The post-intervention vertical separation between the two predicted lines corresponds to the interaction term β₃ (male: 18.263; female: 12.050), visually illustrating the substantive improvement in standing long jump performance associated with the new assessment standard.

To facilitate cross-outcome comparison, we further summarized the standardized effect-size ranges for the three primary outcomes in the male and female subsamples (Table 14). Overall, the effect sizes for intrinsic motivation, extrinsic motivation, and standing long jump performance fell within the moderate-to-large range (*d* ≈ 0.78–0.98). This pattern indicates that the impact of the multidimensional progress-based assessment extends beyond statistical significance and reflects clear practical relevance with interpretable effect magnitudes.

**Table 14:** Standardized effect sizes for DID estimates.

Outcome	Gender	DID (β3)	Cohen’s d_DID	Hedges’ g_DID
Internal motivation	Male	0.86	0.98	0.98
Internal motivation	Female	0.878	0.95	0.94
External motivation	Male	0.803	0.91	0.9
External motivation	Female	0.832	0.83	0.82
Standing long jump	Male	18.263	0.91	0.9
Standing long jump	Female	12.050	0.78	0.78

Building on these results, we formally tested whether the net intervention effects differed by gender (Table 15). The Treat × Post × Gender interaction was not statistically significant for intrinsic motivation, extrinsic motivation, or standing long jump performance (*p* = 0.947, 0.919, and 0.296, respectively), indicating that the current data provide no evidence that the intervention effect varies by gender. Together with the consistent effect directions and comparable effect magnitudes across the three outcomes, these findings suggest that the assessment reform exerted a stable and relatively balanced facilitative effect for students of different genders.

**Table 15:** Gender moderation tests for DID treatment effects.

Outcome	β3_male (SE)	β3_female (SE)	Treat × Post × Gender (=female–male)	95% CI	*p*
Intrinsic motivation	0.860 (0.185)	0.878 (0.201)	0.018	[−0.517, 0.553]	0.947
Extrinsic motivation	0.803 (0.195)	0.832 (0.207)	0.029	[−0.528, 0.586]	0.919
Standing long jump	18.263 (4.609)	12.050 (3.740)	−6.213	[−17.847, 5.421]	0.296

β3_male and β3_female are the DID net effects (Treat × Post) estimated from gender-specific models. Treat × Post × Gender reports the female–male contrast in the DID effects, with 95% confidence intervals and *p*-values.

In summary, the findings from Study 2 support a stable treatment effect of the multidimensional assessment standard at the overall sample level, and no significant gender differences were observed. Given that group assignment was conducted at the class level, and that further stratification by subgroups (e.g., ethnic background) may lead to insufficient statistical power, this study did not estimate ethnicity-related stratified effects or interaction effects. Accordingly, the present inference focuses primarily on the average treatment effect at the population level. Future research could draw on larger samples and multi-institution data to examine heterogeneity across gender and ethnic groups more rigorously, thereby providing a more systematic assessment of equity and external validity.

## 3 Discussion

This study adopted a sequential mixed-methods development-and-evaluation design, following a “development first, quantitative validation second” pathway to evaluate the effects of a multidimensional progress-based assessment standard for standing long jump performance in university public physical education courses. This design is particularly appropriate for research contexts in which an intervention tool or assessment system must first be developed and then empirically tested (Creswell and Clark, 2017). In Study 1, the Delphi method and the analytic hierarchy process (AHP) were used to establish a multidimensional assessment framework and a weighting scheme, thereby providing a structured and implementable assessment standard for the intervention. In Study 2, a class-level cluster-randomized quasi-experimental design was employed, and a DID model was used to estimate the net effect of the assessment reform. Such pre–post intervention designs with intervention and control groups are common in physical education research and help generate practice-relevant evidence under authentic classroom conditions (Vaquero-Solís et al., 2020).

Overall, the results indicate that, relative to the control group, the intervention group achieved substantially greater gains in intrinsic motivation, extrinsic motivation, and standing long jump performance. This pattern suggests that an assessment reform centered on multidimensional and progress-oriented criteria has positive instructional-intervention value. This finding aligns with an international trend in physical education assessment research, in which assessment is shifting from a “single outcome orientation” towards alternative approaches that place greater emphasis on process, feedback, and multidimensional evidence (Slingerland et al., 2024). In public physical education classes, assessment practices shape not only how students’ performance is represented, but also students’ participation quality, practice investment, and attitudes towards the course. Accordingly, assessment reform often carries pedagogical significance beyond its role as a grading tool (López-Pastor et al., 2013). From an implementation perspective, the main costs of the proposed system are concentrated in early-stage teacher training and score standardization. In practice, implementation burden can be reduced through a one-off centralized training session accompanied by standardized scoring sheets. During routine instruction, teachers only need to generate periodic progress feedback according to a fixed schedule. For large-class settings, automated calculation can further improve scalability. Overall, this system is better positioned as a replicable classroom assessment process embedded within existing instruction, rather than as an additional stand-alone testing component. To facilitate comparison with prior intervention studies, this study also reported standardized effect sizes. The results show that the DID-standardized effects for the three primary outcomes fell within the moderate-to-large range, indicating that the assessment reform was not only statistically significant but also substantively meaningful in practice. Accordingly, the findings should be interpreted as a task-specific proof of concept rather than as validation of a comprehensive university physical education assessment system. The main transferable contribution is the workflow—literature-based dimension generation, Delphi consensus, AHP weighting, local data calibration, and classroom evaluation—rather than the specific scores or weights. Education authorities, universities, and sport-science departments can adapt this workflow to develop locally calibrated standards for other fitness components instead of applying external norms without population-specific validation.

From a Self-Determination Theory (SDT) perspective, multidimensional progress-based assessment may enhance students’ learning motivation by supporting their needs for autonomy and competence. SDT posits that when individuals experience stronger autonomy, competence, and relatedness in a learning environment, their motivation is more likely to shift from external regulation to more autonomous forms, accompanied by sustained engagement and more positive affective experiences (Ryan and Deci, 2024). In this study, the multidimensional progress-based evaluation transformed the learning process into a trackable and adjustable goal-attainment pathway by using progress rules referenced to students’ individual baselines, publicly transparent scoring criteria, and periodic formative feedback. This design enabled students to identify their own improvement priorities and adapt their practice strategies, thereby strengthening their sense of autonomy. At the same time, a continuous chain of growth evidence (baseline–progress–outcome), together with an explicit acknowledgement that improvement becomes increasingly difficult as performance rises, helped students perceive a more controllable link between effort and performance gains. This, in turn, reinforced their sense of competence and supported the internalization of motivation. In physical education contexts, psychological need satisfaction shows stable associations with higher autonomous motivation, greater classroom engagement, and more positive learning experiences (White et al., 2021). Compared with traditional “one-off tests” or assessment systems centred on a single summative outcome, the present assessment standard places greater emphasis on process performance and growth magnitude. Through publicly stated criteria and transparent reporting, the system continuously communicates “improvable directions” and “attainable goals” to students. Such informational feedback is more likely to be interpreted as support for competence development rather than as a controlling demand, and is therefore more conducive to maintaining learning interest and motivational stability (Hattie and Timperley, 2007). In assessment contexts, task-improvement-focused information also tends to protect students’ interest and subsequent performance more effectively than normative grading, as supported by classic work in educational psychology (Butler and Nisan, 1986).

It is important to emphasize that the motivational gains observed in this study did not remain at the attitudinal level. Instead, they co-occurred with significant improvements in standing long jump performance, suggesting that the assessment reform may have generated practical performance benefits by fostering greater practice investment and more effective technique refinement. Research on formative assessment indicates that when learners can access actionable feedback information throughout the learning process and compare their current performance with target standards, they are more likely to adjust their learning behaviors effectively (Wiliam, 2011). Feedback research further highlights that high-quality feedback helps learners clarify “where the gap is” and “how to close the gap,” thereby supporting skill optimization and performance improvement (Hattie and Timperley, 2007). In the present study, the assessment system did not merely report a summative score. It also provided students with continuous evidence of growth by linking pretest baselines to staged improvements. This design may help students generate internal feedback and translate assessment information into implementable practice strategies (Nicol, 2021). Moreover, transparent disclosure of criteria and scoring dimensions may strengthen students’ self-monitoring and self-evaluation capacities. Substantial empirical evidence indicates that self-assessment and peer assessment can contribute to improved learning performance (Yan et al., 2022).

At the gender level, the intervention produced significant positive effects for both male and female students, with consistent directions across outcomes. This pattern suggests that the assessment reform has potential applicability across genders, and that, under the current sample and classroom implementation conditions, the system can exert a stable facilitative effect for students of different genders. Prior physical education research also suggests that gender differences may be shaped by socialization experiences, patterns of classroom participation, and sensitivity to feedback. Accordingly, future research remains necessary to examine potential gender-related differences in mechanisms and boundary conditions of assessment reforms (Ntoumanis et al., 2021).

Although the DID results support a significant net gain in the intervention group, causal mechanism interpretations should remain cautious. On the one hand, the control group also showed some natural improvement under non-intervention conditions, which may be attributable to semester-long training progression, familiarity effects from repeated testing, or routine instructional influences. On the other hand, variation in teachers’ implementation, shifts in classroom climate, and students’ reactive responses to being observed or assessed may also influence motivation and performance. Therefore, a more defensible conclusion is that multidimensional progress-based assessment, as an implementable classroom assessment reform tool, demonstrated clear advantages in this setting, while its effects may have emerged from the joint influence of assessment structure and instructional context.

The broader implication of the study concerns process rather than universal norms. Universities, sport-science departments, and education authorities could adapt the same workflow to other fitness components: define the local construct and population, obtain structured expert input, translate the qualitative requirements into measurable dimensions, estimate weights, calibrate conversion functions with local data, and test the resulting standard in practice. This procedure can reduce uncritical reliance on cut scores developed for different populations. However, localisation requires recalibration rather than direct transfer. A standard for endurance, flexibility, speed, or another national or institutional population would need its own expert panel, reference data, validity evidence, and impact evaluation.

The external validity of this study has clear boundaries. The intervention was conducted in public physical education courses at one Chinese university, and performance was measured only through the standing long jump. The resulting standard is neither a national norm nor a comprehensive physical education assessment system. It should be interpreted as preliminary evidence for a task closely related to lower-limb explosive power in comparable university settings. Future studies should repeat the development process for endurance, speed, flexibility, strength, and sport-skill tasks and should examine whether multiple locally validated components can be combined into a broader assessment framework. The two-wave design also precluded a formal pre-trend test and analysis of dynamic change. Multi-wave, multi-institution studies could use multi-period DID or latent growth models and examine heterogeneity across demographic groups. Although class-level allocation and cluster-robust DID estimation strengthened the analysis, teacher implementation, repeated-testing familiarity, and participant reactivity may still have influenced the results. Finally, motivation was self-reported; future research should add behavioral indicators such as attendance, practice records, or wearable-derived activity.

## 4 Conclusion

This study developed and evaluated a four-dimension progress-based assessment standard for standing long jump performance in university physical education. The standard combined baseline performance, end-point attainment, improvement magnitude, and improvement difficulty, and its implementation was associated with greater gains in intrinsic motivation, extrinsic motivation, and standing long jump performance than the conventional assessment approach. The findings support the feasibility of progress-sensitive assessment for this specific fitness task, not a universal standard for physical education. The main contribution is a replicable development-and-evaluation workflow that can be locally recalibrated and independently validated for other fitness components and student populations.

## Data Availability

The raw data supporting the conclusions of this article will be made available by the authors, without undue reservation.
