# Supplementary material for: Developing and evaluating a multidimensional progress-based assessment standard for standing long jump performance in university physical education: a sequential mixed-methods study
Source: Front Psychol. 2026 Aug 12;17:1861330. doi: 10.3389/fpsyg.2026.1861330 (PMC13506395; doi:10.3389/fpsyg.2026.1861330)
Supplement: Supplementary file 1 [file Table_1.DOCX]

Appendix

1. Table A1. Standing Long Jump Scoring Standards
2. Table A2. Men's Standing Long Jump Scoring Standards Breakdown Table (Unit: cm)
3. Table A3. Women's Standing Long Jump Scoring Standards Breakdown Table (Unit: cm)
4. Table A4. Hale T score evaluation
5. Table A5. Progress T-score breakdown table (0-30 points range)
6. Table A6. Progress T-score breakdown table (32.5-60 points range)
7. Table A7. Progress T-score breakdown table (62.5-100 points range)
8. Table A8. Expert AHP results
9. Table A9. Pre-test of each indicator before intervention Descriptive Statistics
10. Table A10. $\text{∆}$ Descriptive Statistics
11. Table A11. 95% CI error bars (±)

Table A1. Standing Long Jump Scoring Standards

| Level | Score | Male  Performance (cm) | Female Performance (cm) |
| --- | --- | --- | --- |
| Excellent | 100 | 273 | 207 |
|  | 95 | 268 | 201 |
|  | 90 | 263 | 195 |
| Good | 85 | 256 | 188 |
|  | 80 | 248 | 181 |
| Pass | 78 | 244 | 178 |
|  | 76 | 240 | 175 |
|  | 74 | 236 | 172 |
|  | 72 | 232 | 169 |
|  | 70 | 228 | 166 |
|  | 68 | 224 | 163 |
|  | 66 | 220 | 160 |
|  | 64 | 216 | 157 |
|  | 62 | 212 | 154 |
|  | 60 | 208 | 151 |
| Fail | 50 | 203 | 146 |
|  | 40 | 198 | 141 |
|  | 30 | 193 | 136 |
|  | 20 | 188 | 131 |
|  | 10 | 183 | 126 |

Table A2. Men's Standing Long Jump Scoring Standards Breakdown Table (Unit: cm)

| Score | Performance | Score | Performance | Score | Performance | Score | Performance |
| --- | --- | --- | --- | --- | --- | --- | --- |
| 100 | 273 | 80 | 249 | 68 | 225 | 46 | 201 |
| 99 | 272 | 80 | 248 | 68 | 224 | 44 | 200 |
| 98 | 271 | 79 | 247 | 67 | 223 | 42 | 199 |
| 97 | 270 | 79 | 246 | 67 | 222 | 40 | 198 |
| 96 | 269 | 78 | 245 | 66 | 221 | 38 | 197 |
| 95 | 268 | 78 | 244 | 66 | 220 | 36 | 196 |
| 94 | 267 | 77 | 243 | 65 | 219 | 34 | 195 |
| 93 | 266 | 77 | 242 | 65 | 218 | 32 | 194 |
| 92 | 265 | 76 | 241 | 64 | 217 | 30 | 193 |
| 91 | 264 | 76 | 240 | 64 | 216 | 28 | 192 |
| 90 | 263 | 75 | 239 | 63 | 215 | 26 | 191 |
| 89 | 262 | 75 | 238 | 63 | 214 | 24 | 190 |
| 88 | 261 | 74 | 237 | 62 | 213 | 22 | 189 |
| 88 | 260 | 74 | 236 | 62 | 212 | 20 | 188 |
| 87 | 259 | 73 | 235 | 61 | 211 | 18 | 187 |
| 86 | 258 | 73 | 234 | 61 | 210 | 16 | 186 |
| 86 | 257 | 72 | 233 | 60 | 209 | 14 | 185 |
| 85 | 256 | 72 | 232 | 60 | 208 | 12 | 184 |
| 84 | 255 | 71 | 231 | 58 | 207 | 10 | 183 |
| 84 | 254 | 71 | 230 | 56 | 206 | 8 | 182 |
| 83 | 253 | 70 | 229 | 54 | 205 | 6 | 181 |
| 82 | 252 | 70 | 228 | 52 | 204 | 4 | 180 |
| 82 | 251 | 69 | 227 | 50 | 203 | 2 | 179 |
| 81 | 250 | 69 | 226 | 48 | 202 | 0 | 178 |

Table A3. Women's Standing Long Jump Scoring Standards Breakdown Table (Unit: cm)

| Score | Performance | Score | Performance | Score | Performance | Score | Performance |
| --- | --- | --- | --- | --- | --- | --- | --- |
| 100 | 207 | 83 | 185 | 68 | 163 | 42 | 142 |
| 99 | 206 | 82 | 184 | 67 | 162 | 40 | 141 |
| 98 | 205 | 82 | 183 | 67 | 161 | 38 | 140 |
| 97 | 204 | 81 | 182 | 66 | 160 | 36 | 139 |
| 97 | 203 | 80 | 181 | 65 | 159 | 34 | 138 |
| 96 | 202 | 79 | 180 | 65 | 158 | 32 | 137 |
| 95 | 201 | 79 | 179 | 64 | 157 | 30 | 136 |
| 94 | 200 | 78 | 178 | 63 | 156 | 28 | 135 |
| 93 | 199 | 77 | 177 | 63 | 155 | 26 | 134 |
| 93 | 198 | 77 | 176 | 62 | 154 | 24 | 133 |
| 92 | 197 | 76 | 175 | 61 | 153 | 22 | 132 |
| 91 | 196 | 75 | 174 | 61 | 152 | 20 | 131 |
| 90 | 195 | 75 | 173 | 60 | 151 | 18 | 130 |
| 89 | 194 | 74 | 172 | 60 | 151 | 16 | 129 |
| 88 | 193 | 73 | 171 | 58 | 150 | 14 | 128 |
| 88 | 192 | 73 | 170 | 56 | 149 | 12 | 127 |
| 87 | 191 | 72 | 169 | 54 | 148 | 10 | 126 |
| 86 | 190 | 71 | 168 | 52 | 147 | 8 | 125 |
| 86 | 189 | 71 | 167 | 50 | 146 | 6 | 124 |
| 85 | 188 | 70 | 166 | 48 | 145 | 4 | 123 |
| 84 | 187 | 69 | 165 | 46 | 144 | 2 | 122 |
| 84 | 186 | 69 | 164 | 44 | 143 | 0 | 121 |

Tabe A4. Hale T score evaluation

| Score | 30 | 35 | 40 | 45 | 50 | 55 | 60 | 65 | 70 | 75 | 80 | 85 | 90 | 95 | 100 |
| --- | --- | --- | --- | --- | --- | --- | --- | --- | --- | --- | --- | --- | --- | --- | --- |
| 95～99 | -24 | -22 | -20 | -18 | -16 | -14 | -12 | -10 | -8 | -6 | -4 | -2 | 0 | 2 | 4 |
| 90～94 | -22 | -20 | -18 | -16 | -14 | -12 | -10 | -8 | -6 | -4 | -2 | 0 | 2 | 4 | 6 |
| 85～89 | -20 | -18 | -16 | -14 | -12 | -10 | -8 | -6 | -4 | -2 | 0 | 2 | 4 | 6 | 8 |
| 80～84 | -18 | -16 | -14 | -12 | -10 | -8 | -6 | -4 | -2 | 0 | 2 | 4 | 6 | 8 | 10 |
| 75～79 | -16 | -14 | -12 | -10 | -8 | -6 | -4 | -2 | 0 | 2 | 4 | 6 | 8 | 10 | 12 |
| 70～74 | -14 | -12 | -10 | -8 | -6 | -4 | -2 | 0 | 2 | 4 | 6 | 8 | 10 | 12 | 14 |
| 65～69 | -12 | -10 | -8 | -6 | -4 | -2 | 0 | 2 | 4 | 6 | 8 | 10 | 12 | 14 | 16 |
| 60～64 | -10 | -8 | -6 | -4 | -2 | 0 | 2 | 4 | 6 | 8 | 10 | 12 | 14 | 16 | 18 |
| 55～59 | -8 | -6 | -4 | -2 | 0 | 2 | 4 | 6 | 8 | 10 | 12 | 14 | 16 | 18 | 20 |
| 50～54 | -6 | -4 | -2 | 0 | 2 | 4 | 6 | 8 | 10 | 12 | 14 | 16 | 18 | 20 | 22 |
| 45～49 | -4 | -2 | 0 | 2 | 4 | 6 | 8 | 10 | 12 | 14 | 16 | 18 | 20 | 22 | 24 |
| 40～44 | -2 | 0 | 2 | 4 | 6 | 8 | 10 | 12 | 14 | 16 | 18 | 20 | 22 | 24 | 26 |
| 35～39 | 0 | 2 | 4 | 6 | 8 | 10 | 12 | 14 | 16 | 18 | 20 | 22 | 24 | 26 | 28 |
| 30～34 | 2 | 4 | 6 | 8 | 10 | 12 | 14 | 16 | 18 | 20 | 22 | 24 | 26 | 28 | 30 |
| 25～29 | 4 | 6 | 8 | 10 | 12 | 14 | 16 | 18 | 20 | 22 | 24 | 26 | 28 | 30 | 32 |
| 20～24 | 6 | 8 | 10 | 12 | 14 | 16 | 18 | 20 | 22 | 24 | 26 | 28 | 30 | 32 | 34 |
| 15～19 | 8 | 10 | 12 | 14 | 16 | 18 | 20 | 22 | 24 | 26 | 28 | 30 | 32 | 34 | 36 |
| 10～14 | 10 | 12 | 14 | 16 | 18 | 20 | 22 | 24 | 26 | 28 | 30 | 32 | 34 | 36 | 38 |
| 5～9 | 12 | 14 | 16 | 18 | 20 | 22 | 24 | 26 | 28 | 30 | 32 | 34 | 36 | 38 | 40 |
| 0～4 | 14 | 16 | 18 | 20 | 22 | 24 | 26 | 28 | 30 | 32 | 34 | 36 | 38 | 40 | 42 |

Table A5. Progress T-score breakdown table (0-30 points range)

| Score | 0 | 2.5 | 5 | 7.5 | 10 | 12.5 | 15 | 17.5 | 20 | 22.5 | 25 | 27.5 | 30 |
| --- | --- | --- | --- | --- | --- | --- | --- | --- | --- | --- | --- | --- | --- |
| 95-99 | -36 | -35 | -34 | -33 | -32 | -31 | -30 | -29 | -28 | -27 | -26 | -25 | -24 |
| 90-94 | -34 | -33 | -32 | -31 | -30 | -29 | -28 | -27 | -26 | -25 | -24 | -23 | -22 |
| 85-89 | -32 | -31 | -30 | -29 | -28 | -27 | -26 | -25 | -24 | -23 | -22 | -21 | -20 |
| 80-84 | -30 | -29 | -28 | -27 | -26 | -25 | -24 | -23 | -22 | -21 | -20 | -19 | -18 |
| 75-79 | -28 | -27 | -26 | -25 | -24 | -23 | -22 | -21 | -20 | -19 | -18 | -17 | -16 |
| 70-74 | -26 | -25 | -24 | -23 | -22 | -21 | -20 | -19 | -18 | -17 | -16 | -15 | -14 |
| 65-69 | -24 | -23 | -22 | -21 | -20 | -19 | -18 | -17 | -16 | -15 | -14 | -13 | -12 |
| 60-64 | -22 | -21 | -20 | -19 | -18 | -17 | -16 | -15 | -14 | -13 | -12 | -11 | -10 |
| 55-59 | -20 | -19 | -18 | -17 | -16 | -15 | -14 | -13 | -12 | -11 | -10 | -9 | -8 |
| 50-54 | -18 | -17 | -16 | -15 | -14 | -13 | -12 | -11 | -10 | -9 | -8 | -7 | -6 |
| 45-49 | -16 | -15 | -14 | -13 | -12 | -11 | -10 | -9 | -8 | -7 | -6 | -5 | -4 |
| 40-44 | -14 | -13 | -12 | -11 | -10 | -9 | -8 | -7 | -6 | -5 | -4 | -3 | -2 |
| 35-39 | -12 | -11 | -10 | -9 | -8 | -7 | -6 | -5 | -4 | -3 | -2 | -1 | 0 |
| 30-34 | -10 | -9 | -8 | -7 | -6 | -5 | -4 | -3 | -2 | -1 | 0 | 1 | 2 |
| 25-29 | -8 | -7 | -6 | -5 | -4 | -3 | -2 | -1 | 0 | 1 | 2 | 3 | 4 |
| 20-24 | -6 | -5 | -4 | -3 | -2 | -1 | 0 | 1 | 2 | 3 | 4 | 5 | 6 |
| 15-19 | -4 | -3 | -2 | -1 | 0 | 1 | 2 | 3 | 4 | 5 | 6 | 7 | 8 |
| 10-14 | -2 | -1 | 0 | 1 | 2 | 3 | 4 | 5 | 6 | 7 | 8 | 9 | 10 |
| 5-9 | 0 | 1 | 2 | 3 | 4 | 5 | 6 | 7 | 8 | 9 | 10 | 11 | 12 |
| 0-4 | 2 | 3 | 4 | 5 | 6 | 7 | 8 | 9 | 10 | 11 | 12 | 13 | 14 |

Table A6. Progress T-score breakdown table (32.5-60 points range)

| Score | 32.5 | 35 | 37.5 | 40 | 42.5 | 45 | 47.5 | 50 | 52.5 | 55 | 57.5 | 60 |
| --- | --- | --- | --- | --- | --- | --- | --- | --- | --- | --- | --- | --- |
| 95～99 | -23 | -22 | -21 | -20 | -19 | -18 | -17 | -16 | -15 | -14 | -13 | -12 |
| 90～94 | -21 | -20 | -19 | -18 | -17 | -16 | -15 | -14 | -13 | -12 | -11 | -10 |
| 85～89 | -19 | -18 | -17 | -16 | -15 | -14 | -13 | -12 | -11 | -10 | -9 | -8 |
| 80～84 | -17 | -16 | -15 | -14 | -13 | -12 | -11 | -10 | -9 | -8 | -7 | -6 |
| 75～79 | -15 | -14 | -13 | -12 | -11 | -10 | -9 | -8 | -7 | -6 | -5 | -4 |
| 70～74 | -13 | -12 | -11 | -10 | -9 | -8 | -7 | -6 | -5 | -4 | -3 | -2 |
| 65～69 | -11 | -10 | -9 | -8 | -7 | -6 | -5 | -4 | -3 | -2 | -1 | 0 |
| 60～64 | -9 | -8 | -7 | -6 | -5 | -4 | -3 | -2 | -1 | 0 | 1 | 2 |
| 55～59 | -7 | -6 | -5 | -4 | -3 | -2 | -1 | 0 | 1 | 2 | 3 | 4 |
| 50～54 | -5 | -4 | -3 | -2 | -1 | 0 | 1 | 2 | 3 | 4 | 5 | 6 |
| 45～49 | -3 | -2 | -1 | 0 | 1 | 2 | 3 | 4 | 5 | 6 | 7 | 8 |
| 40～44 | -1 | 0 | 1 | 2 | 3 | 4 | 5 | 6 | 7 | 8 | 9 | 10 |
| 35～39 | 1 | 2 | 3 | 4 | 5 | 6 | 7 | 8 | 9 | 10 | 11 | 12 |
| 30～34 | 3 | 4 | 5 | 6 | 7 | 8 | 9 | 10 | 11 | 12 | 13 | 14 |
| 25～29 | 5 | 6 | 7 | 8 | 9 | 10 | 11 | 12 | 13 | 14 | 15 | 16 |
| 20～24 | 7 | 8 | 9 | 10 | 11 | 12 | 13 | 14 | 15 | 16 | 17 | 18 |
| 15～19 | 9 | 10 | 11 | 12 | 13 | 14 | 15 | 16 | 17 | 18 | 19 | 20 |
| 10～14 | 11 | 12 | 13 | 14 | 15 | 16 | 17 | 18 | 19 | 20 | 21 | 22 |
| 5～9 | 13 | 14 | 15 | 16 | 17 | 18 | 19 | 20 | 21 | 22 | 23 | 24 |
| 0～4 | 15 | 16 | 17 | 18 | 19 | 20 | 21 | 22 | 23 | 24 | 25 | 26 |

Table A7. Progress T-score breakdown table (62.5-100 points range)

| Score | 62.5 | 65 | 67.5 | 70 | 72.5 | 75 | 77.5 | 80 | 82.5 | 85 | 87.5 | 90 | 92.5 | 95 | 97.5 | 100 |
| --- | --- | --- | --- | --- | --- | --- | --- | --- | --- | --- | --- | --- | --- | --- | --- | --- |
| 95～99 | -11 | -10 | -9 | -8 | -7 | -6 | -5 | -4 | -3 | -2 | -1 | 0 | 1 | 2 | 3 | 4 |
| 90～94 | -9 | -8 | -7 | -6 | -5 | -4 | -3 | -2 | -1 | 0 | 1 | 2 | 3 | 4 | 5 | 6 |
| 85～89 | -7 | -6 | -5 | -4 | -3 | -2 | -1 | 0 | 1 | 2 | 3 | 4 | 5 | 6 | 7 | 8 |
| 80～84 | -5 | -4 | -3 | -2 | -1 | 0 | 1 | 2 | 3 | 4 | 5 | 6 | 7 | 8 | 9 | 10 |
| 75～79 | -3 | -2 | -1 | 0 | 1 | 2 | 3 | 4 | 5 | 6 | 7 | 8 | 9 | 10 | 11 | 12 |
| 70～74 | -1 | 0 | 1 | 2 | 3 | 4 | 5 | 6 | 7 | 8 | 9 | 10 | 11 | 12 | 13 | 14 |
| 65～69 | 1 | 2 | 3 | 4 | 5 | 6 | 7 | 8 | 9 | 10 | 11 | 12 | 13 | 14 | 15 | 16 |
| 60～64 | 3 | 4 | 5 | 6 | 7 | 8 | 9 | 10 | 11 | 12 | 13 | 14 | 15 | 16 | 17 | 18 |
| 55～59 | 5 | 6 | 7 | 8 | 9 | 10 | 11 | 12 | 13 | 14 | 15 | 16 | 17 | 18 | 19 | 20 |
| 50～54 | 7 | 8 | 9 | 10 | 11 | 12 | 13 | 14 | 15 | 16 | 17 | 18 | 19 | 20 | 21 | 22 |
| 45～49 | 9 | 10 | 11 | 12 | 13 | 14 | 15 | 16 | 17 | 18 | 19 | 20 | 21 | 22 | 23 | 24 |
| 40～44 | 11 | 12 | 13 | 14 | 15 | 16 | 17 | 18 | 19 | 20 | 21 | 22 | 23 | 24 | 25 | 26 |
| 35～39 | 13 | 14 | 15 | 16 | 17 | 18 | 19 | 20 | 21 | 22 | 23 | 24 | 25 | 26 | 27 | 28 |
| 30～34 | 15 | 16 | 17 | 18 | 19 | 20 | 21 | 22 | 23 | 24 | 25 | 26 | 27 | 28 | 29 | 30 |
| 25～29 | 17 | 18 | 19 | 20 | 21 | 22 | 23 | 24 | 25 | 26 | 27 | 28 | 29 | 30 | 31 | 32 |
| 20～24 | 19 | 20 | 21 | 22 | 23 | 24 | 25 | 26 | 27 | 28 | 29 | 30 | 31 | 32 | 33 | 34 |
| 15～19 | 21 | 22 | 23 | 24 | 25 | 26 | 27 | 28 | 29 | 30 | 31 | 32 | 33 | 34 | 35 | 36 |
| 10～14 | 23 | 24 | 25 | 26 | 27 | 28 | 29 | 30 | 31 | 32 | 33 | 34 | 35 | 36 | 37 | 38 |
| 5～9 | 25 | 26 | 27 | 28 | 29 | 30 | 31 | 32 | 33 | 34 | 35 | 36 | 37 | 38 | 39 | 40 |
| 0～4 | 27 | 28 | 29 | 30 | 31 | 32 | 33 | 34 | 35 | 36 | 37 | 38 | 39 | 40 | 41 | 42 |

Table A8. A Expert AHP results

| Dimension (A) | A_1_ | A_2_ | A_3_ | A_4_ |
| --- | --- | --- | --- | --- |
| Pre-test score (A1) | 1 | 1/5 | 1/3 | 1/3 |
| Post-test score (A2) | 5 | 1 | 3 | 3 |
| Progress (A3) | 3 | 1/3 | 1 | 1 |
| Progressive Difficulty (A4) | 3 | 1/3 | 1 | 1 |

The judgement matrix can be written as：

A**=**$\left[ \begin{matrix} 1 & 1/5 & \begin{matrix} 1/3 & 1/3 \end{matrix} \\ 5 & 1 & \begin{matrix} 3 & 3 \end{matrix} \\ \begin{matrix} 3 \\ 3 \end{matrix} & \begin{matrix} 1/3 \\ 1/3 \end{matrix} & \begin{matrix} \begin{matrix} 1 & 1 \end{matrix} \\ \begin{matrix} 1 & 1 \end{matrix} \end{matrix} \end{matrix} \right]$

After normalization:

$\bar{A}$**=**$\left[ \begin{matrix} 0.083 & 0.107 & \begin{matrix} 0.064 & 0.064 \end{matrix} \\ 0.429 & 0.532 & \begin{matrix} 0.559 & 0.560 \end{matrix} \\ \begin{matrix} 0.245 \\ 0.243 \end{matrix} & \begin{matrix} 0.183 \\ 0.178 \end{matrix} & \begin{matrix} \begin{matrix} 0.188 & 0.188 \end{matrix} \\ \begin{matrix} 0.188 & 0.188 \end{matrix} \end{matrix} \end{matrix} \right]$

Adding up the elements row by row:

$\bar{WA}$**=**$\left[ \begin{matrix} 0.319 \\ 2.080 \\ \begin{matrix} 0.804 \\ 0.797 \end{matrix} \end{matrix} \right]$

After normalization:

WA**=**$\left[ \begin{matrix} 0.080 \\ 0.520 \\ \begin{matrix} 0.201 \\ 0.199 \end{matrix} \end{matrix} \right]$

Table A9. Pre-test of each indicator before intervention Descriptive Statistics

| Gender | Group | N | Index | Skewness Statistic | Kurtosis Statistic |
| --- | --- | --- | --- | --- | --- |
| Male | CG | 67 | Internal motivation | -0.080 | -1.335 |
|  |  |  | External Motivation | -0.129 | -1.175 |
|  |  |  | Standing long jump results | -0.430 | 1.070 |
|  | IG | 72 | Internal motivation | -0.636 | -0.424 |
|  |  |  | External Motivation | -0.445 | -0.684 |
|  |  |  | Standing long jump results | -0.614 | 1.089 |
| Female | CG | 65 | Internal motivation | -0.037 | -1.121 |
|  |  |  | External Motivation | -0.404 | -1.131 |
|  |  |  | Standing long jump results | 0.687 | -0.233 |
|  | IG | 69 | Internal motivation | -0.234 | -1.113 |
|  |  |  | External Motivation | -0.256 | -1.273 |
|  |  |  | Standing long jump results | 0.990 | 1.334 |

Table A10. $\Delta$ Descriptive Statistics

| Gender | Group | N | Index | Skewness Statistic | Kurtosis Statistic |
| --- | --- | --- | --- | --- | --- |
| Male | CG | 67 | Internal motivation | 0.934 | 0.945 |
|  |  |  | External Motivation | 1.093 | 0.495 |
|  |  |  | Standing long jump results | 0.266 | -0.293 |
|  | IG | 72 | Internal motivation | 0.500 | -0.463 |
|  |  |  | External Motivation | 0.670 | 0.045 |
|  |  |  | Standing long jump results | -0.259 | -0.087 |
| Female | CG | 65 | Internal motivation | 0.725 | 0.099 |
|  |  |  | External Motivation | 1.358 | 1.345 |
|  |  |  | Standing long jump results | 0.111 | -0.605 |
|  | IG | 69 | Internal motivation | 0.036 | -1.147 |
|  |  |  | External Motivation | -0.055 | -1.087 |
|  |  |  | Standing long jump results | 0.469 | 0.780 |

Table A11. 95% CI error bars (±)

|  | Internal motivatio | | External motivatio | | Standing long jump | |
| --- | --- | --- | --- | --- | --- | --- |
|  | Male | Female | Male | Female | Male | Female |
| CG-Pre | ±0.242 | ±0.231 | ±0.231 | ±0.237 | ±4.75 | ±3.87 |
| CG-Post | ±0.219 | ±0.218 | ±0.208 | ±0.192 | ±4.36 | ±3.68 |
| IG-Pre | ±0.169 | ±0.214 | ±0.187 | ±0.245 | ±4.71 | ±3.49 |
| IG-Post | ±0.092 | ±0.091 | ±0.124 | ±0.099 | ±4.08 | ±3.52 |
